# Evaluation of General Synthesis Procedures for Bioflavonoid–Metal Complexes in Air-Saturated Alkaline Solutions

**DOI:** 10.3389/fchem.2020.00589

**Published:** 2020-08-05

**Authors:** Yuanyong Yao, Meng Zhang, Laibing He, Yunyang Wang, Shixue Chen

**Affiliations:** Tongren Key Laboratory for Modernization Research, Development and Utilization of Traditional Chinese Medicine and National Medicine, School of Material and Chemical Engineering, Tongren University, Tongren, China

**Keywords:** dihydromyricetin, flavonoids, bioflavonoid–metal complexes, alkaline conditions, superoxide anion, HPLC, UV-visible spectrophotometer, transition metal ions

## Abstract

The general synthesis methods of bioflavonoid–metal complexes are considered to be unreliable due to the instability of flavonoids in air-saturated alkaline solutions. In this study, dihydromyricetin (DHM), as a representative bioflavonoid, was selected for complexation with various transition metal ions in an air-saturated alkaline solution to form DHM–metal(II) complexes, following the general synthetic procedure. After characterization, the metal complexes were hydrolyzed to observe the stability of DHM under acidic conditions via HPLC. The effects of synthetic conditions (metal ion, alkalinity, and reflux time) on DHM stability were then investigated by UV-vis spectroscopy and HPLC. Finally, using electron paramagnetic resonance, DHM and its analogs were observed with DMPO (5,5-dimethyl-1-pyrroline-N-oxide) to form a relatively stable free radical adduct. Multiple peaks corresponding to unknown compounds appeared in the LC spectra of the DHM–metal(II) complexes after hydrolysis, indicating that some DHM reacted during synthesis. Subsequently, the transition metal ion and solution alkalinity were found to have notable effects on the stability of free DHM. Furthermore, DHM and several of its analogs generated the superoxide-anion radical in air-saturated alkaline solutions. Their capacities for generating the superoxide anion seemed to correspond to the number and/or location of hydroxyl groups or their configurations. Interestingly, DHM can react with the superoxide anion to transform into myricetin, which involves the abstraction of a C3–H atom from DHM by O_2_^−^. Therefore, the general synthetic procedure for bioflavonoid–metal complexes in air-saturated alkaline solutions should be improved.

## Introduction

**Figure 1:**
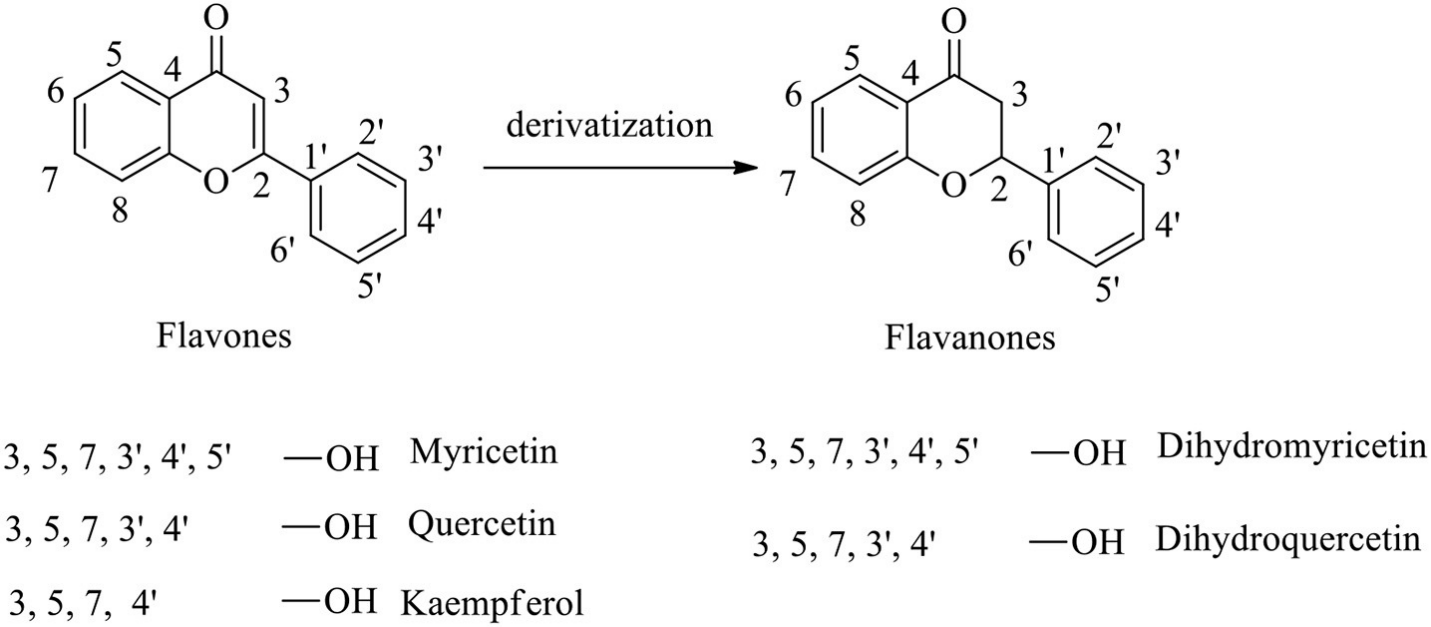
Molecular structures of flavones, flavanones, and their derivatives.

Bioflavonoids are naturally occurring phenolic substances that can be isolated from a wide range of vascular plants, with more than 8,000 individual compounds currently identified. They have been widely used as potent bioactive agents in biological and pharmaceutical fields (Raffa et al., 2017; Spagnuolo et al., 2018; Dai et al., 2019). Recently, numerous studies have revealed that bioflavonoids with novel inherent molecular skeletons [e.g., dihydromyricetin, dihydroquercetin, myricetin, quercetin, kaempferol, and so on (Figure 1)] show valuable biological activities, including anticancer (Raffa et al., 2017; Madunić et al., 2018; Imran et al., 2019), antiviral (Lani et al., 2016; Dai et al., 2019; Lalani and Poh, 2020), anti-inflammatory (Zhang and Tsao, 2016; Chen et al., 2017; Spagnuolo et al., 2018), and other effects (Amirhossein and Shadboorestan, 2016; Liang et al., 2019).

**Figure 2:**
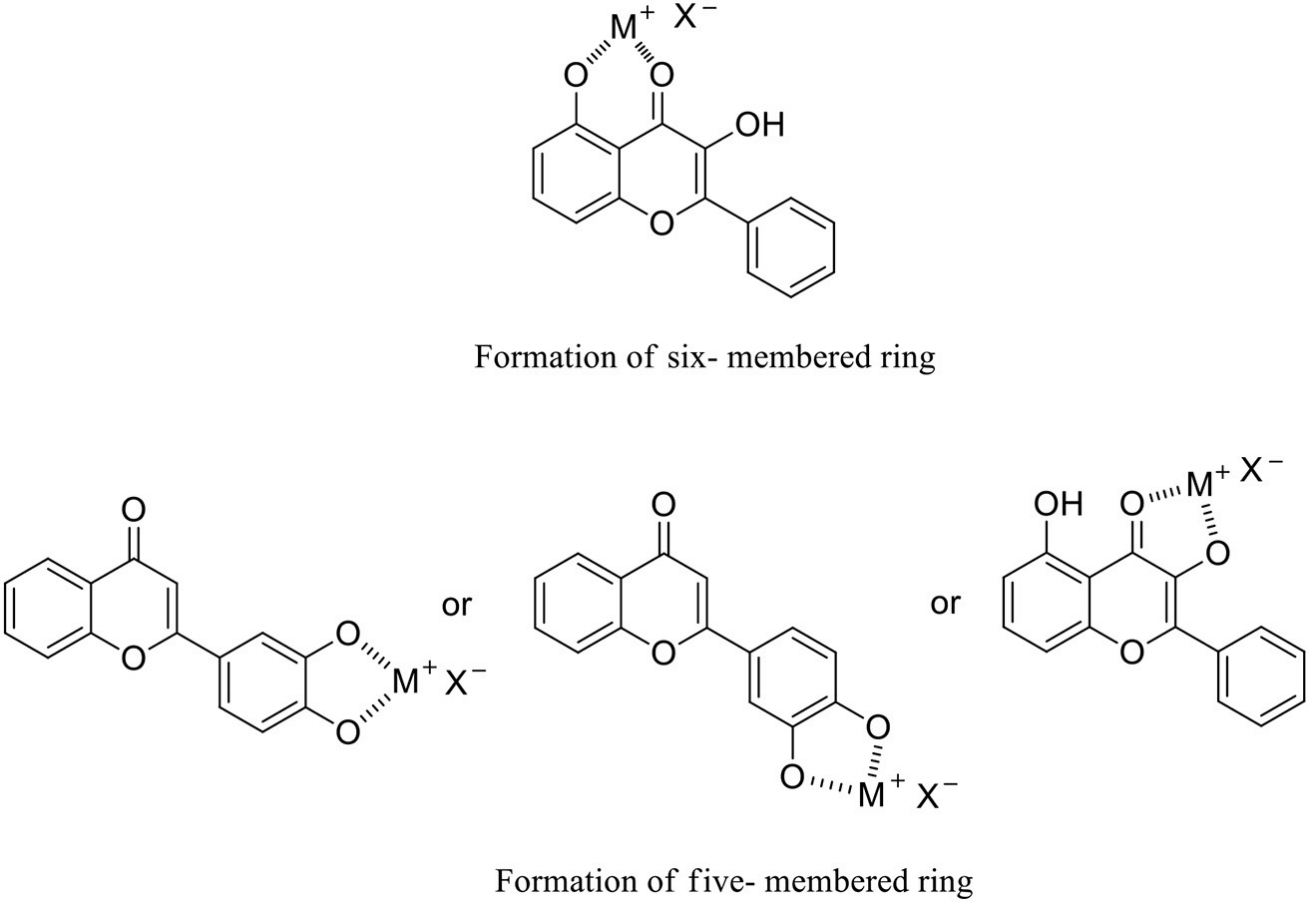
Flavanone–metal complexes via the formation of five- and/or six-membered rings.

To further improve bioflavonoids in terms of their bioactivities, metal complexes with bioflavonoids as ligands to chelate transition metal ions have gained significant scientific interest (Thangavel et al., 2018; Gençkal et al., 2020; Silva et al., 2020; Wu et al., 2020). These complexes form five- and/or six-membered rings through the interaction of the metal ion with the hydroxyl moiety and/or single carbonyl group on the bioflavonoid skeleton (Figure 2). Therefore, approaches to synthesizing bioflavonoid–metal complexes with novel structures are highly desirable.

For instance, metal(II) complexes with quercetin (Gençkal et al., 2020; Silva et al., 2020), hesperitin (Tamayo et al., 2016), kaempferol (Tu et al., 2016; Thangavel et al., 2018), myricetin (Uivarosi et al., 2019), and dihydromyricetin (Li et al., 2016; Wu et al., 2020) as representative examples have been synthesized by following a general synthetic procedure for bioflavonoid–metal complexes (Tu et al., 2016; Maitera et al., 2018; Thangavel et al., 2018; Khater et al., 2019; Gençkal et al., 2020). The general procedure involves the deprotonation of the phenolic hydroxyl group(s) of the bioflavonoid into the corresponding phenolate, which then chelates the metal ion in an air-saturated alkaline solution. These metal complexes have been characterized by FT-IR and UV-vis spectroscopy, elemental analysis, X-ray analyses, and so on. Furthermore, their potential bioactivities have been observed as somewhat higher than that of the corresponding original bioflavonoids in terms of antioxidant capacity (Samsonowicz et al., 2017; Khater et al., 2019), anticancer effects (Qian et al., 2015; Khater et al., 2019), and superoxide anion/hydroxyl free radical scavenging (Jabeen et al., 2017).

In our previous work on bioflavonoid stabilization, we found via UV-vis spectroscopy that most bioflavonoids are unstable in air-saturated alkaline solutions, which prompted us to reevaluate the general synthetic procedure for bioflavonoid–metal complexes. To address this, in this study, dihydromyricetin (DHM) was selected as a representative bioflavonoid to prepare DHM–M(II) complexes using the general procedure. Subsequently, the metal complexes were identified by FT-IR and UV-vis spectroscopy, elemental analysis, and evaluation of antioxidant capacity. Finally, the DHM–M(II) complexes were hydrolyzed to observe any changes in their molecular structures under acidic conditions by means of high-performance liquid chromatography (HPLC). The results indicated that the air-saturated alkaline solution used in the general synthetic procedure is not suitable for the synthesis of bioflavonoid–metal complexes. Moreover, to determine the probable causes of the observed issue with the general synthetic procedure, the synthetic conditions (e.g., metal ion, alkalinity, and reflux time) affecting DHM stabilization were discussed.

## Experimental Section

### Materials and Synthesis

All of the reagents are analytical grade or chromatographically pure, and were purchased from commercial sources. All solutions were prepared using double-distilled water. Dihydromyricetin (DHM) was extracted by our laboratory from tender stems and leaves of Ampelsis grossedentata with more than 95% purity. Standard DHM was purchased from Aladdin Reagent, Shanghai (China). Metal ion reagents (copper, zinc, iron, nickel, and cobalt acetates) were also bought from Aladdin Reagent, Shanghai (China). Organic solvents were obtained from Kemiou reagent, Tianjin (China).

### General DHM–M(II) Synthetic Procedure

According to the literature (Tu et al., 2016; Maitera et al., 2018; Thangavel et al., 2018; Khater et al., 2019; Gençkal et al., 2020), the general synthetic procedure for DHM–M(II) complexes is as follows: solid DHM (0.5820 g, 1.82 mmol) was dissolved completely in ethanol (5 mL, 95%, W/W). Then, the mixture was transferred to 50 mL of an alkaline solution (sodium acetate dissolved in a water–ethanol solution (50%, v/v) at pH = 8.2) under stirring for 5 min. Afterward, metal ion reagents (2 mmol) (copper, zinc, iron, nickel, and cobalt acetates) were separately dissolved in ethanol (5 mL, 95%, W/W) and added to the above mixture under reflux for 120 min. The reaction mixture was cooled to room temperature, precipitated, and filtered, and the resulting solid was washed three times with EtOH and double-distilled water in turn. Subsequently, after re-crystallization in acetone, the solid product was dried under vacuum for 48 h at −20°C to afford the DHM–M(II) complexes.

## Instrumentation

EPR spectra were recorded at room temperature, utilizing a Bruker A300 spectrometer for detection of superoxide anion using a center magnetic field at 3500.00 G, sweep width at 150.00 G, microwave power at 3.99 mW, modulation amplitude at 1.000 G, with scanning field and switching time operating for 30 s and 40 ms, respectively. The UV-vis spectra were measured on a UV-759S UV-visible spectrophotometer (Jingke, China). The IR spectroscopies were recorded as KBr pellets on an IR Affinity-1S infrared spectrometer in the frequency range 400–4,000 cm^−1^. The HPLC was recorded on a LC-20AT spectrometer, equipped with a DAD detector (Shimadzu, Japan). The NMR spectra were recorded on a JEOL-ECX 500 instrument (500 MHz for ^1^H, 125 MHz for ^13^C) using DMSO-d_6_ as the solvent. Tetramethylsilane (δ = 0) and DMSO-d_6_ (δ = 40.1) served as internal standards for ^1^H NMR and ^13^C NMR spectral experiments, respectively. The mass spectral analysis was performed on Waters XevoTQ-XSMs Apparatus. The element analysis (Flash Smart, Thermo Fisher, Germany) was used to identify compound composition.

### Evaluation of Antioxidant Activity by Pyrogallol Autoxidation

#### Control Group

Na_2_EDTA (1 mM) was added to a Tris-HCl buffer (0.05 M, pH = 8.2, 2.90 mL) solution, followed by the addition of 100 μL of a pyrogallol solution (6 mg in 1 mM HCl). After shaking vigorously for 30 min at room temperature, the resulting mixture was measured by UV-vis spectrophotometer from 200 to 800 nm. The resulting spectra were regarded as the control group.

#### Testing Group

Samples of the DHM–M(II) complexes were prepared at 0.01 mg/mL in DMSO, and free DHM as a positive control was prepared at 0.01 mg/mL in ethanol. Then, 100 μL of each sample was transferred to the above control group sample and shaken vigorously for 1 min. The reaction mixture was then prepared and measured according to the same procedure as for the control group.

ΔA325nm,controlT−ΔA325nm,sampleTΔA325nm,controlT ×100%

ΔA_325nm, control_: absorbance of control group at 325 nm

ΔA_325nm, sample_: absorbance of testing group at 325 nm

T: 25°C.

### Hydrolyzing DHM–M(II) Complexes Under Acidic Conditions for HPLC Analysis

The DHM–M(II) complexes (0.9050 g) were each added to an acidic solution (20 mL, 1 mL HCl in 100 mL of ethanol, pH = 2) and stirred for 3 h at 50°C under a nitrogen atmosphere. Afterward, the reaction mixtures were moderated to pH = 5–6 by 0.1% aqueous NaOH. After filtering and evaporation, the residues were dissolved in chromatographic methanol and subjected to HPLC analysis.

#### Chromatographic Conditions

Hypersil BDS C18 (4.6^*^200 mm, 5 um), Elute: (Acetonitrile / Water (0.1 % phosphoric acid) = 24 / 76), Flow rate = 1 ml/min, UV = 254 nm.

### Assessment of Conditions Affecting the Stability of Free DHM

#### Reflux Time

Solid DHM was dissolved in a water–ethanol solution (50%, v/v) at a concentration of 0.05 mg/mL, and 10 mL was refluxed for different periods. After cooling to room temperature, all samples were subjected to UV-vis analysis.

#### Metal Ion (Ni^2+^, Zn^2+^, Cu^2+^, Co^2+^, Fe^2+^)

A solution (10 mL) of 0.05 mg/mL DHM in water–ethanol (50%, v/v) was combined with each of the metal ion reagents (0.0016 mmol, 0.5 mL in water–ethanol solution) under stirring for 120 min at 25°C. The reaction mixtures were then measured by UV-vis spectroscopy.

#### Alkalinity

A 1.0 mg/mL solution of DHM (0.5 mL) was added to 10 mL of a sodium acetate solution (pH = 8.2) under stirring for 1, 3, 5, 10, 20, and 30 min at room temperature. Afterward, the reaction mixtures were subjected to UV-vis analysis.

#### Alkalinity Investigated Quantitatively by HPLC

Solutions at pH = 7.4, 8.2, and 9.4 were prepared by dissolving sodium acetate/acetic acid in water–ethanol (50%, v/v). Of a 0.8 mg/mL DHM solution, 1 mL was added to 10 mL of each alkaline solution with stirring for several minutes at room temperature. The resulting mixtures were moderated to weak acidity (approximately pH = 6) with 0.1% HCl. The control group was prepared in the same manner without the alkaline solution. After evaporating the solvent under vacuum, the test and control groups were dissolved in 1 mL of methanol and filtered (0.45 μm). Finally, the samples were subjected to HPLC for quantitative analysis.

**Chromatographic conditions**: Hypersil BDS C18 (4.6^*^200 mm, 5 um), Elute: (Acetonitrile / Water (0.1 % phosphoric acid) = 24 / 76), Flow rate = 1 ml/min, UV = 254 nm.

### Separation and Purification of Myricetin (MYR) as an Oxidized Product of DHM in Air-Saturated Alkaline Solution (pH = 8.2)

A 100 mg/mL DHM solution was prepared, to which 3–20 mL of an alkaline solution (pH = 8.2) was added, and the reaction mixture was stirred continuously. After 2 h, the mixture was quenched with 0.1% HCl to pH = 5–6. After evaporating the solvent under vacuum, the residue was dissolved in ethanol and dried over MgSO_4_. Subsequently, after filtering and evaporation of the solvent under vacuum, the solution was evaporated to dryness. The residue was separated and purified by column chromatography on silica gel (EtOAc/petroleum ether from 1/25 to 1/3) to afford product **A** (50.2 mg) as a yellow solid. ^1^H-NMR (500 MHz, DMSO-D_6_, 25°C, TMS): δ (ppm) 12.51 (s, 1H), 10.82 (s, 1H), 9.36 (s, 1H), 9.25 (s, 2H), 8.83 (s, 1H), 7.25 (s, 2H), 6.38 (s, 1H), 6.19 (s, 1H); ^13^C-NMR (125 MHz, DMSO-D_6_, 25°C, TMS): δ (ppm) 176.21, 164.31, 161.17, 156.52, 147.27, 146.16, 136.31, 121.23, 107.60, 103.42, 98.61, 93.66. Anal. Calcd for C_15_H_10_O_8_: C, 56.61; H, 3.17; O, 40.22; Found: C, 56.45; H, 3.24. ESI-MS m/z (CH_3_OH): 318.04; Found: 358 [M+H+K]^+^.

A solution of product **A** at 1.0 mg/mL was prepared and subjected to HPLC analysis. **Chromatographic conditions**: Hypersil BDS C18 (4.6^*^200 mm, 5 um), Elute: (Acetonitrile / Water (0.1% phosphoric acid) = 24 / 76), Flow rate = 1 ml/min, UV = 254 nm.

### Superoxide Anion From DHM in Air-Saturated Alkaline Solution

Using an air supplier, air, nitrogen, and oxygen were continuously forced into separate alkaline solutions (pH = 8.2) for 10 min. DHM was dissolved in methanol to 0.20 mg/mL, and 100 μL was added to 1 mL of each of the air-, oxygen-, and nitrogen-saturated alkaline solutions with continuous stirring for 1 min. To 100 μL of those solutions, 5,5-dimethyl-1-pyrroline-N-oxide (DMPO) (100 mg/L in methanol, 10 μL) was added, and the resulting mixtures were transferred into silica tubes and subjected to EPR analysis.

### Superoxide Anions Generated by the DMSO System

Dry DMSO (1 mL) was transferred to a test tube (5 mL), and oxygen was introduced to the system for 10 min at room temperature using an air supplier. Sodium phenolate (100 mmol/L, 100 μL) was immediately added to the system and stirred slowly for 30 min at 37°C under an oxygen atmosphere. DMPO (100 mg/L in methanol, 10 μL) was added to 100 μL of this solution, and the resulting mixture was transferred into a silica tube and subjected to EPR analysis.

### Transformation of DHM Into MYR by Superoxide-Anion Radicals

To the above described DMSO system, a DHM solution (1.0 mg/mL in dry DMSO) was added with shaking for ~5 min. When the solution changed from colorless to yellow, it was quenched with saturated aqueous NH_4_Cl. After filtering (0.45 μm), the solution was subjected to HPLC analysis using the above chromatographic conditions.

### DHM Analogs Autoxidized in Air-Saturated Alkaline Solution and Observed by EPR

Following the above conditions for the DHM generation of superoxide anions, DHM analogs (MYR, quercetin, daidzein, genistein, chrysin, baicalein, rutin hydrate, and kaempferol) were each prepared at the same concentration (2.0 mg/mL). The solutions were transferred to an air-saturated alkaline solution (1 mL, pH = 8.2) with continuous stirring for 1 min. To 100 μL of these solutions DMPO (100 mg/L in methanol, 10 μL) was added. The resulting mixtures were transferred to silica tubes and subjected to EPR analysis.

## Results and Discussion

### Antioxidant Activity of DHM–M(II) Complexes

By following the reported general synthetic procedure (Tu et al., 2016; Maitera et al., 2018; Thangavel et al., 2018; Khater et al., 2019; Gençkal et al., 2020), the DHM–M(II) complexes DHM–Co(II), DHM–Cu(II), DHM–Fe(II), DHM–Zn(II), and DHM–Ni(II) were obtained. The metal complexes were characterized by FT-IR, elemental analysis, UV-vis, and antioxidant capacity. The IR absorption frequency of the functional groups attached to the DHM–M(II) complexes were red-shifted compared with those of free DMY, indicating the complexation of DHM with metal ions (Supplementary Figure 1 and Supplementary Table 1). Elemental analysis demonstrated that the DHM–metal(II) complexes had the expected compositions (Supplementary Table 2). The UV-vis spectra showed red-shifts and changes in intensity of the absorption of the complexes, further evidencing the complexation of DHM with the metal ions under the selected experimental conditions (Supplementary Figure 2). Finally, the antioxidant activities of the DHM–M(II) complexes were confirmed as higher than that of DHM at the same concentration (Supplementary Figure 3 and Supplementary Table 3). Therefore, it is reasonable to assume that the prepared DHM–M(II) complexes are consistent with those in previous studies.

### Hydrolysis of DHM–M(II) Complexes

To further understand whether DHM changes during complexation, DHM and its metal complexes were hydrolyzed under the same acidic conditions. After stirring for 3 h at 50°C, the resulting mixtures were subjected to HPLC with a monitoring wavelength of 254 nm. In the LC spectra, we found that free DHM stirred in an acidic solution was stable, without any impurity peaks. However, in the spectra of the DHM–M(II) complexes, many unknown absorption peaks with different intensities appeared (Supplementary Figure 4). This caused us to question the general synthetic procedure for bioflavonoid–metal complexes. Subsequently, to address this, the synthetic conditions of the metal ion, alkalinity, and reflux time were investigated by UV-vis or HPLC to observe their influence on the stability of free DHM.

### Effect of Synthesis Conditions on Stability of Free DHM

**Figure 3:**
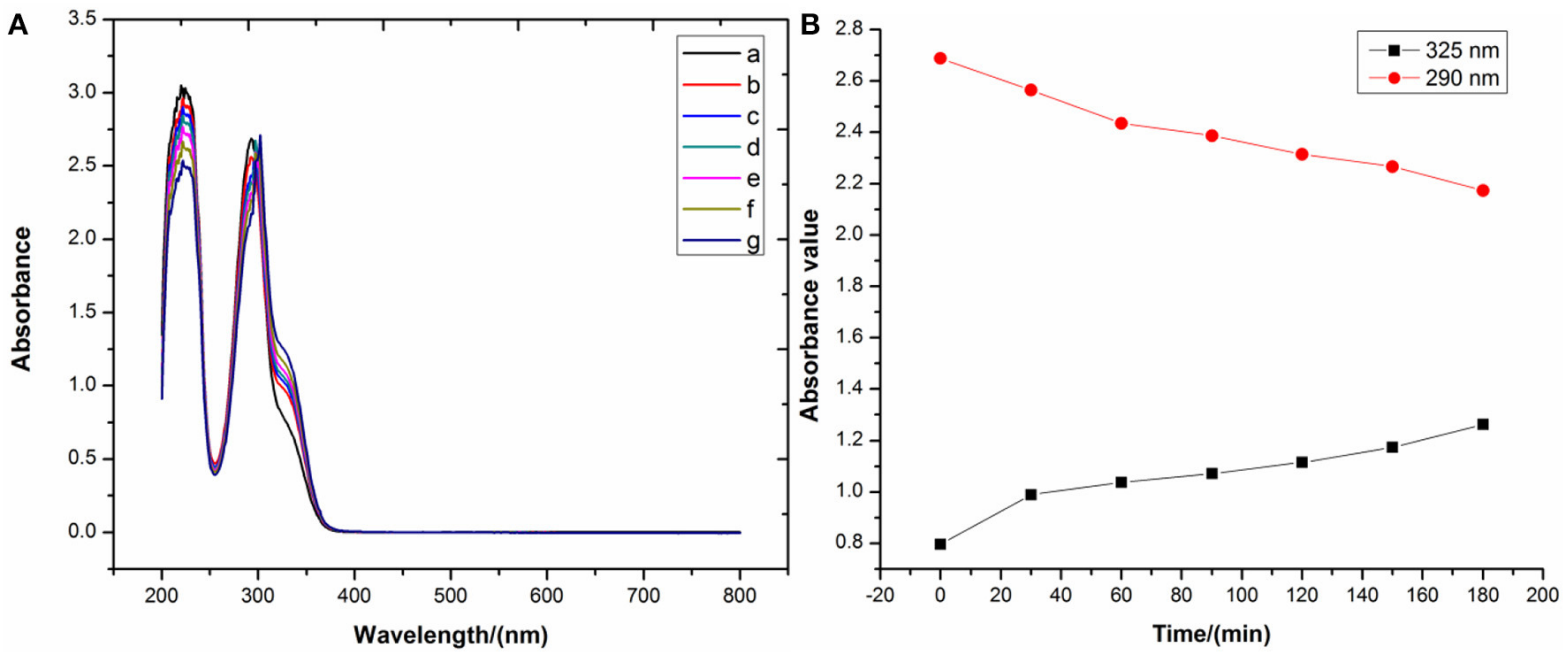
**(A)** UV-vis spectra and **(B)** main peak positions of DHM refluxed for different times.

Reflux time was first investigated by refluxing free DHM for different times in a water–ethanol solution (v/v, 50%) followed by observation by UV-vis spectroscopy. In the resulting spectra, no notable shift in the DHM absorption peak at 325 nm was observed after refluxing for several minutes compared with free DHM without any workup (Figure 3). This indicated that reflux time does not significantly affect the stability of free DHM in an aqueous solution in the absence of alkalinity or metal ions. Therefore, we did not consider reflux time to be a crucial factor affecting the synthesis of the DHM–metal complexes.

**Figure 4:**
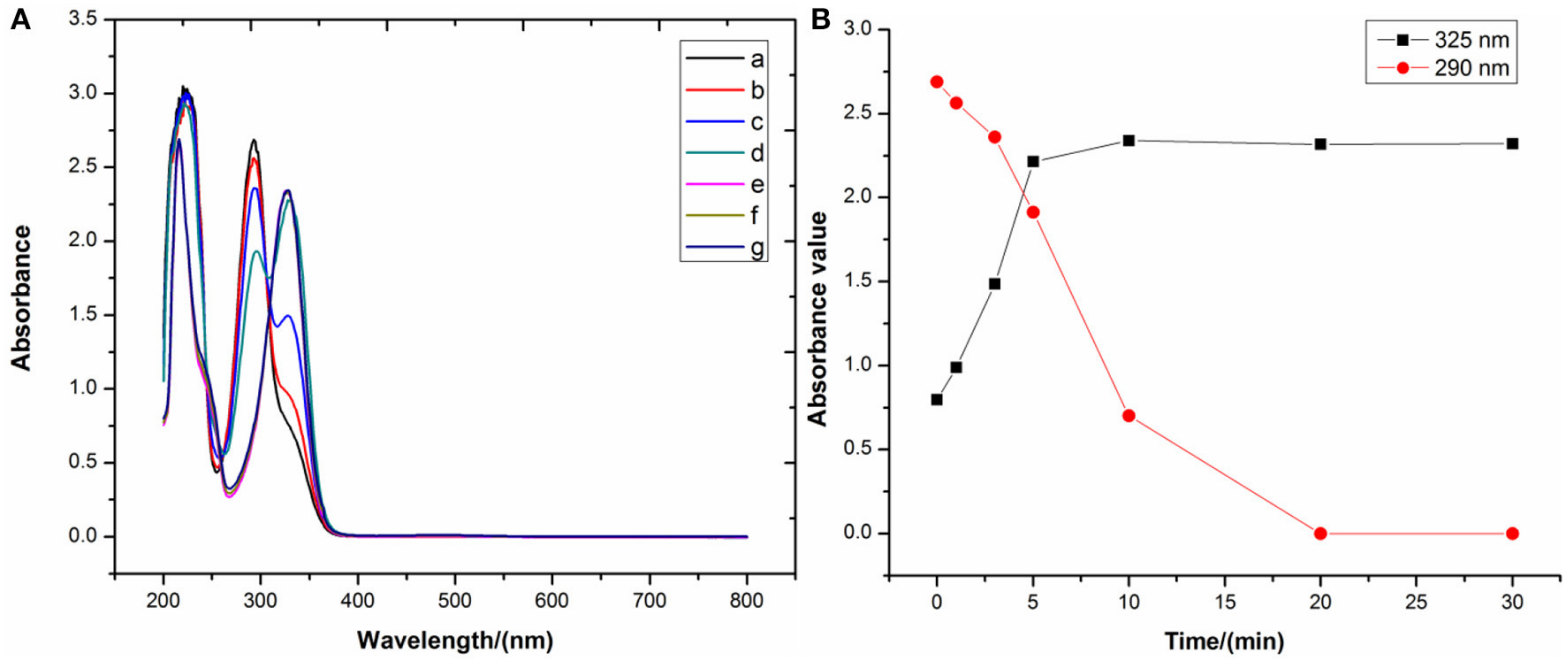
**(A)** UV-vis spectra and **(B)** main peak positions of DHM stirred for different times at pH = 8.2.

To the best of our knowledge, bioflavonoids are generally weakly acidic in ethanol or aqueous solutions, relying on their phenolic hydroxyl moieties to ionize in the liquid phase. In the general synthetic procedure for bioflavonoid–metal complexes, alkalinity promotes the deprotonation of the bioflavonoid phenolic hydroxyl groups to form the corresponding phenolate. Since phenolates can easily donate a pair of electrons, they are better ligands to chelate with metal ions. Thus, alkalinity seems to be a dominant factor in synthesizing DHM–M(II) complexes. A pH of 8.2 for the reaction solution was selected to investigate the influence of alkalinity on free DHM by UV-vis spectroscopy. With longer stirring times at room temperature, the absorbance at 290 nm shifted to 325 nm and increased in intensity (Figure 4), implying that dissolving DHM in an air-saturated alkaline solution decreased the amount of DHM over time. Therefore, the effect of alkalinity on DHM stabilization required further investigation.

**Figure 5:**
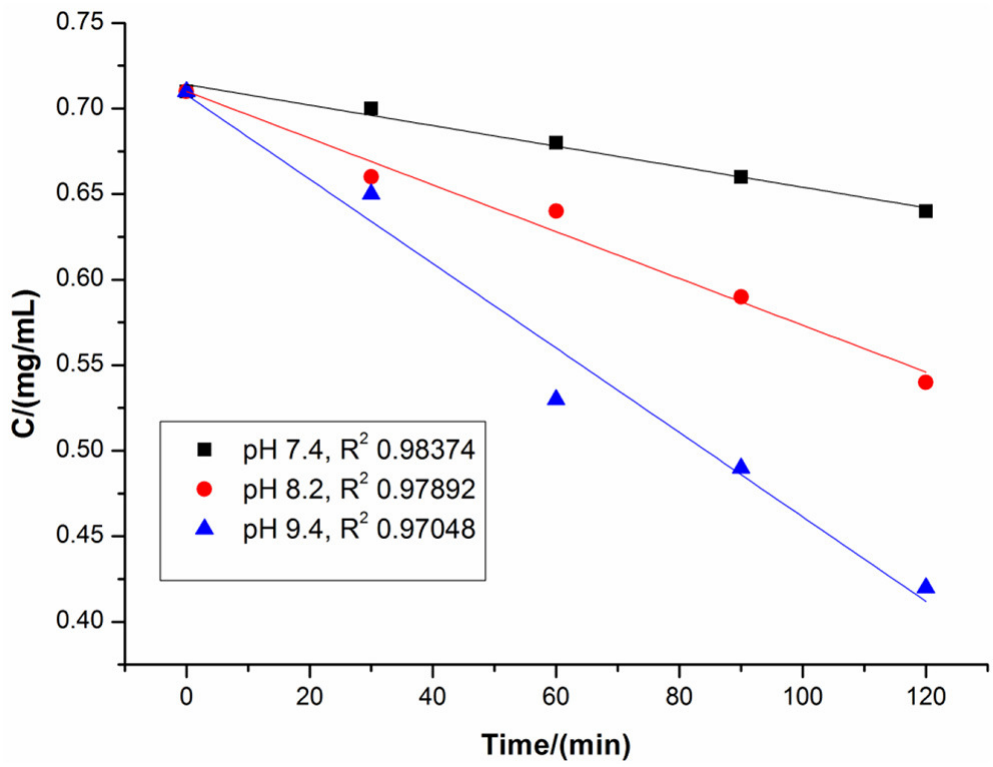
Concentration of DHM depending on alkalinity.

HPLC as a quantitative and qualitative analysis method was first utilized to investigate the effect of alkalinity on free DHM stabilization (Supplementary Figures 5–8 and Supplementary Tables 4–6). As shown in Figure 5, the air-saturated alkaline solution was adjusted to a pH of 7.4 by sodium acetate/acetic acid, and free DHM was stirred for different times in the solution at room temperature. The resulting mixtures were quenched with 0.1% HCl to approximately pH = 6 and subjected to HPLC. We found that the concentration of DHM decreased from 0.71 to 0.64 mg/mL after stirring for 120 min. When the solution pH was moderated to 8.2, the DHM concentration decreased more than at pH = 7.4; similarly, the concentration decrease was the most severe for pH = 9.4. This confirmed the alkalinity as a significant factor in the synthesis of the DHM–M(II) complexes.

**Figure 6:**
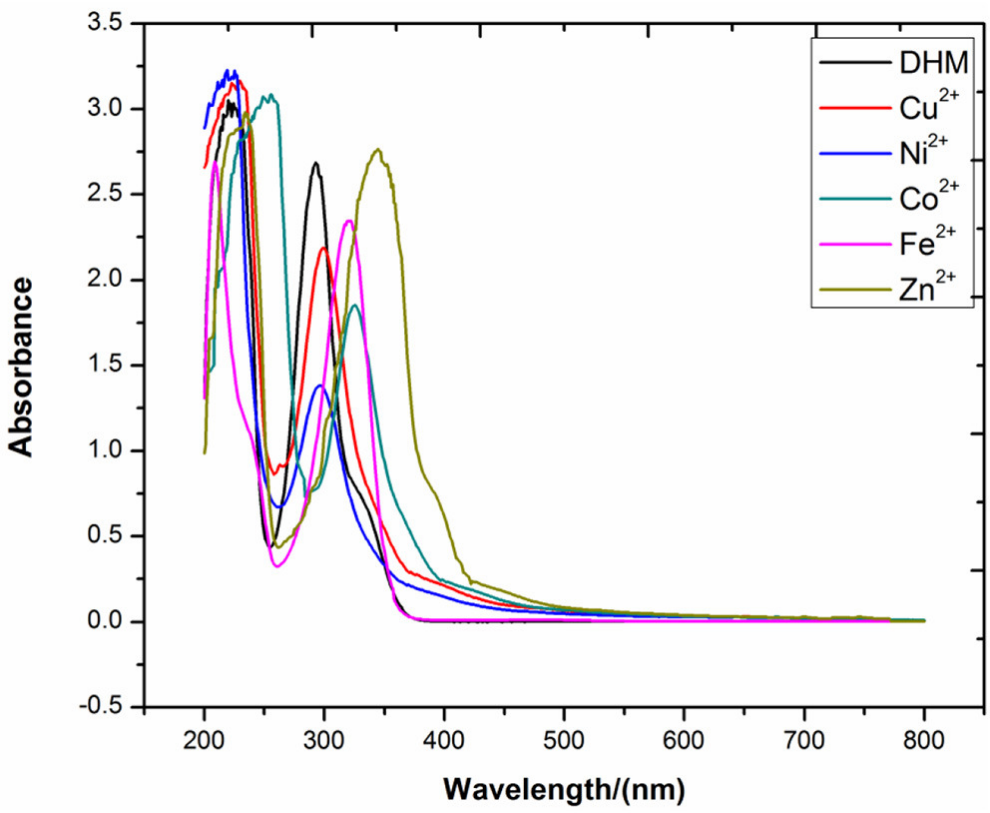
UV-vis spectra of DHM with different metal ions.

The metal ion is an important participant in the synthesis of the DHM–M(II) complexes as it supplies empty orbitals for complexation with the ligand. Importantly, most transition metal ions in an electron-loss state are electronegative and are thus generally considered to be oxidants. In addition, bioflavonoids are electron-rich and thus easily lose electrons after deprotonation of their phenolic hydroxyl moieties. The presence of electronegative metal ions with the phenolates derived from deprotonated bioflavonoids can initiate a redox reaction. Therefore, the effect of metal ions with different electronegativities on DHM stabilization deserves further consideration. DHM was stirred for 2 h in the presence of the different metal ions without alkalinity at room temperature, and the resulting mixtures were subjected to UV-vis analysis. As shown in Figure 6, the intensity and position of the DHM absorption peak were affected by the metal ion. With Zn^2+^, the DHM peak red-shifted from 290 to 345 nm, indicating the effective complexation of Zn^2+^ with free DHM. In contrast, Ni^2+^, Cu^2+^, Co^2+^, and Fe^2+^ not only red-shifted the peak at 290 nm to different extents but also changed the intensity, suggesting that the redox of free DHM also occurred. These results showed that the metal ions affect the DHM stability according to their electronegativity in the order of Ni^2+^ > Co^2+^ > Cu^2+^ > Fe^2+^ > Zn^2+^. Therefore, the metal ion is a dominant factor affecting the stability of DHM in DHM–M(II) complexes.

In summary, synthesizing bioflavonoid–metal complexes in air-saturated alkaline solutions is rational in theory. However, in reality, both the alkalinity and metal ions as important participants in the synthesis of DHM–M(II) complexes were shown to be probable reasons for the lack of stability.

### Transformation of DHM Into MYR in the Presence of Superoxide-Anion Radical

**Scheme 1:**
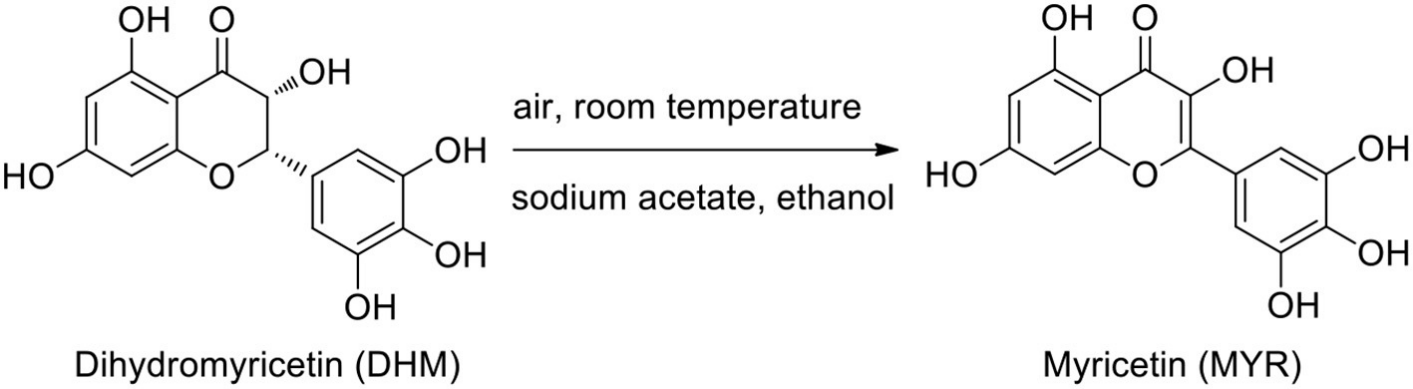
Transformation of DHM into MYR in an alkaline solution.

Based on the above results regarding the synthetic factors affecting DHM stabilization, it was notable that alkalinity is likely the main factor for the general synthesis of bioflavonoid–metal complexes. In the general synthetic procedure, free DHM was first stirred for different times in an air-saturated alkaline solution (pH = 8.2) for deprotonation into the corresponding phenolate, and the resulting solution was subjected to HPLC. The intensity of the absorption peak (*t* =15.7 min) at 254 nm increased with stirring time (Supplementary Figure 7). Subsequently, the absorption peak was separated by column chromatography with an eluent of P/E (from 25/1 to 3/1) and identified by ^1^H- and ^13^C-NMR, ESI-MS, elemental analysis, and HPLC (Supplementary Figures 9, 10). Interestingly, the component was found to be MYR. As far as we know, MYR is the oxidized product of DHM. Thus, the transformation of DHM into MYR in an air-oxidized alkaline solution required further consideration (Scheme 1).

**Figure 7:**
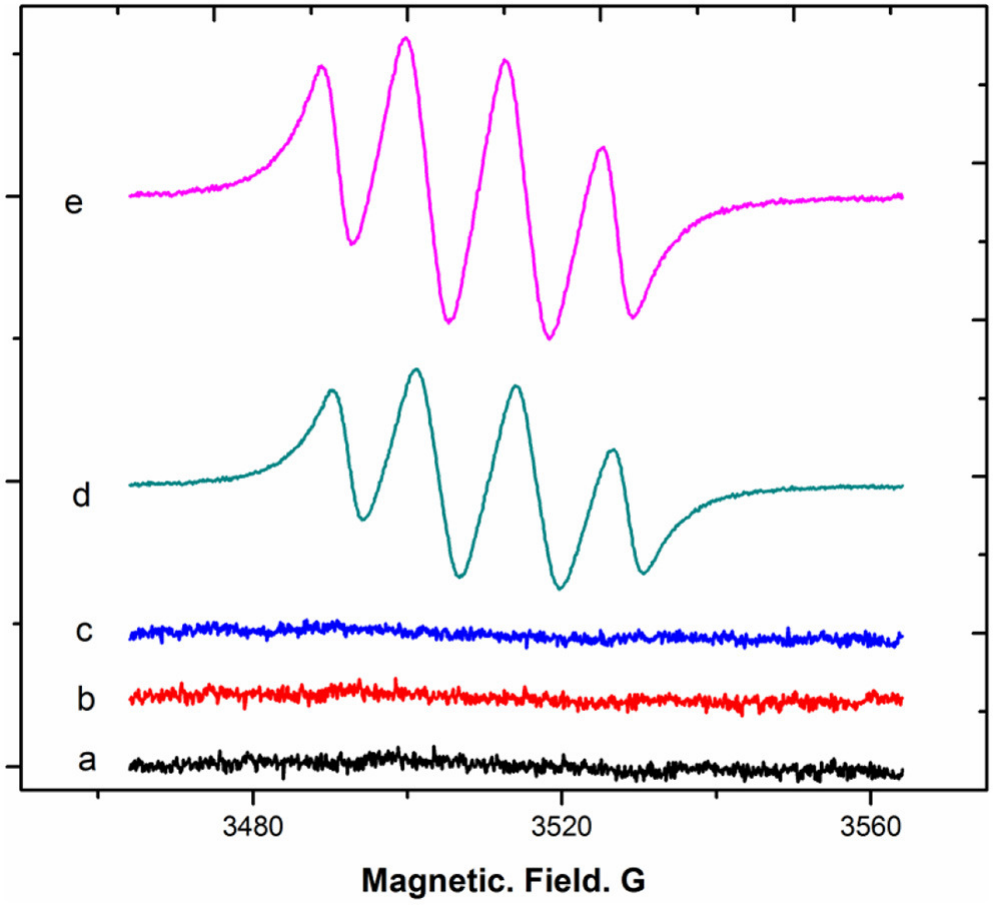
EPR spectra of DHM in air-, nitrogen-, and oxygen-saturated alkaline solutions.

For the transformation of DHM into MYR, DHM after deprotonation by a base forms its electron-rich phenolate, which undergoes a redox reaction upon exposure to an oxidant. We noted that oxygen molecules from the air can dissolve into the alkaline solution. The collision of diatomic oxygen with the phenolate can promote the univalent reduction (single electron transfer) of diatomic oxygen, resulting in one filled and one half-filled antibonding π^*^2p molecular orbitals. This ultimately forms a radical. To validate our proposition, electron paramagnetic resonance (EPR) was applied to observe the radicals generated from DHM dissolved in an air-saturated alkaline (pH = 8.2) solution with DMPO as a spin-trapping electron reagent. DHM dissolved in oxygen- and nitrogen-saturated alkaline solutions at the same pH was also observed for comparison. The EPR results demonstrated that the intensity of the typical signals of the superoxide anion adduct (DMPO-OO–) of DHM in an air-saturated alkaline solution was less than that of the oxygen-saturated alkaline solution, with an overlapping “doublet of a doublet” arising from hyperfine interactions with ^14^N (Liu et al., 1996). When DHM was dissolved in a nitrogen-saturated alkaline solution, no typical adduct signals were observed (Figure 7). Thus, DHM dissolved in an alkaline solution in the presence of oxygen molecules generates superoxide-anion radicals (O_2_^−^) via univalent reduction.

**Figure 8:**
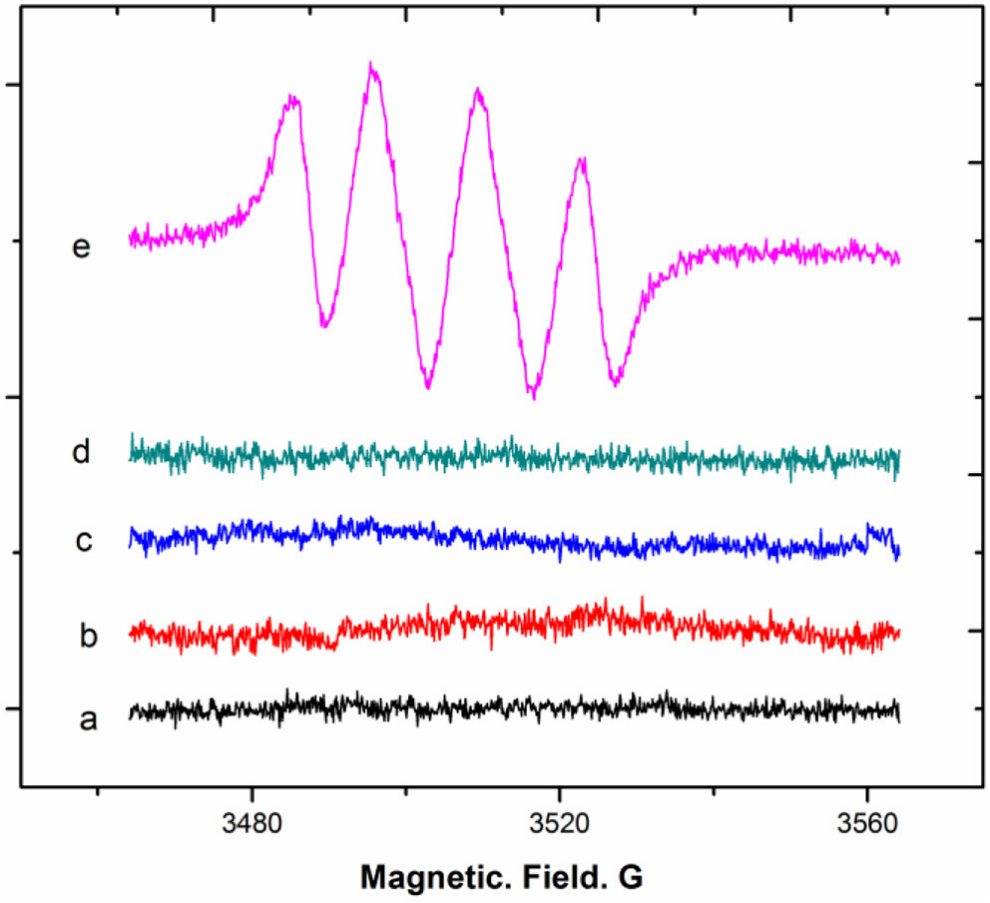
EPR spectra of superoxide-anion radical generation from DMSO reacted with a base in the presence of oxygen.

Next, whether the transformation of DHM into MYR in an air-saturated alkaline solution is associated with O_2_^−^ generation was considered. A system of dry DMSO reacted with sodium phenolate in the presence of oxygen molecules to generate O_2_^−^ was selected according to a previous report (Branchini et al., 2015). The DMSO system was analyzed by EPR with the aid of DMPO. The typical signals of the DMPO-OO–adduct derived from the DMSO system are shown below (Figure 8).

**Scheme 2:**
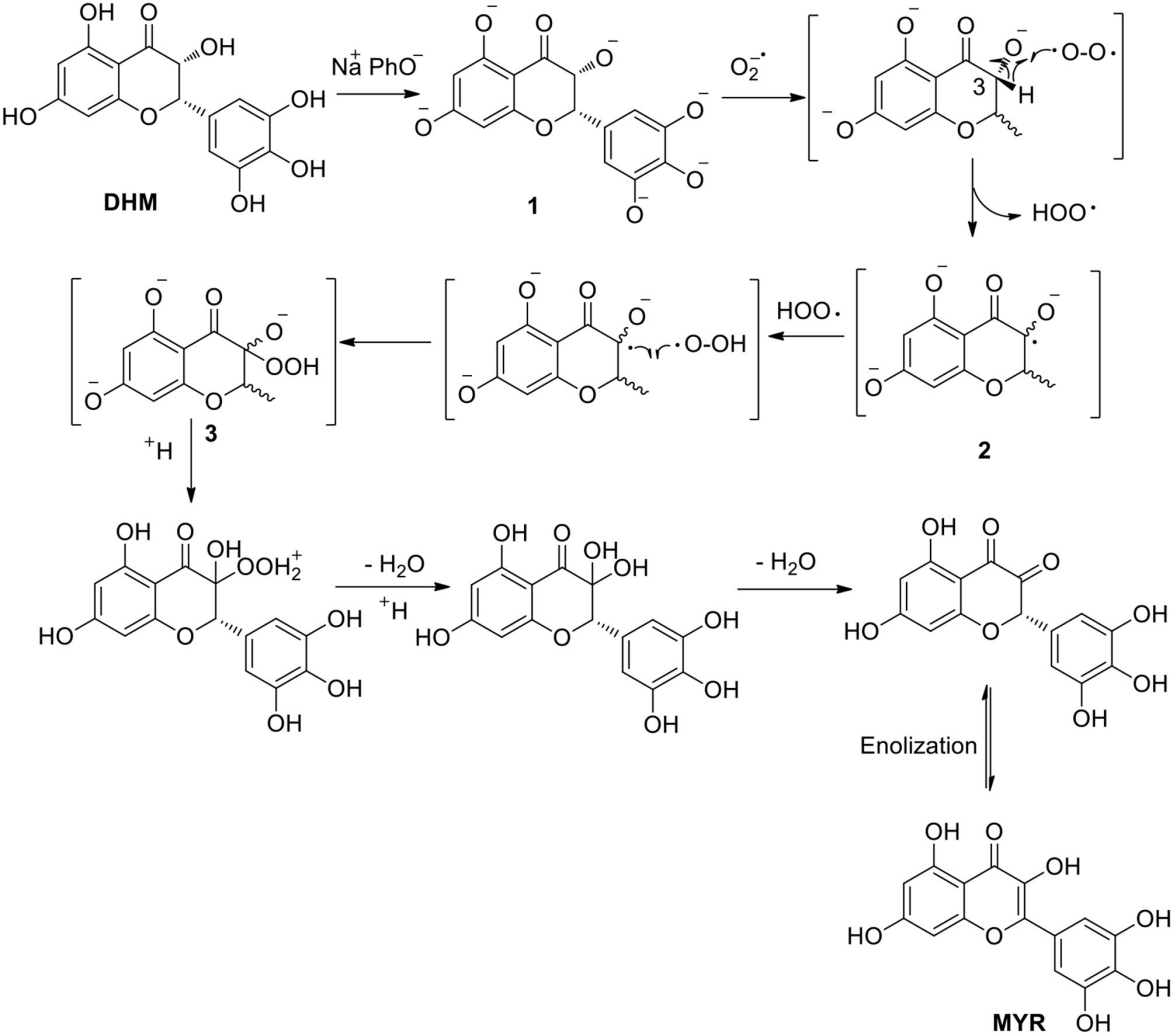
Plausible mechanism of DHM transformation into MYR.

Subsequently, DHM was added to this system of generated superoxide anions with full shaking. Within a few minutes, the color of the resulting solution gradually changed from colorless to yellow. After quenching with a 0.1% HCl solution, the resulting mixture was subjected to HPLC with MYR as a standard for comparison (Supplementary Figure 11). As a result, the compound in the reaction mixture was identified as MYR from the reaction of DHM with superoxide-anion radicals (O_2_^−^). Therefore, we proposed a pathway of DHM transformation into MYR that involves the abstraction of a C3–H atom of phenolate **1** abstracted by O_2_^−^ to form the potent radical **2**. Phenolate **1** was obtained by the deprotonation of the phenolic hydroxyl moieties of DHM. Subsequently, peroxide **3** was generated from the combination of radical **2** with peroxyl radical HOO·. Then, under acidic conditions, terminal product MYR was formed by successive acidification, dehydration, and enolization (Branchini et al., 2015) (Scheme 2).

### Generation of Superoxide-Anion Radical by Other Bioflavonoids as Ligands for Metal Complexes

**Figure 9:**
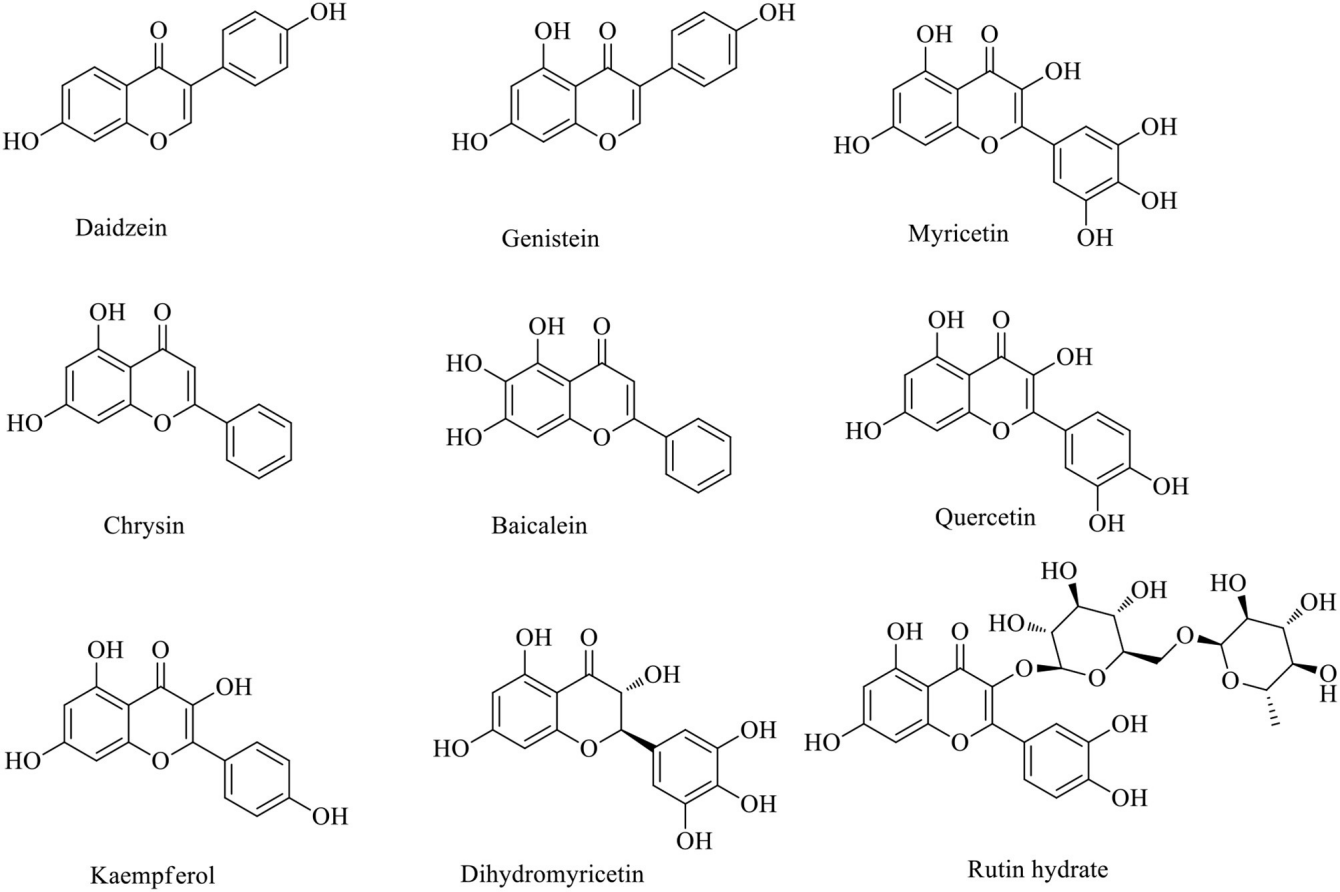
Molecular structures of DHM analogs.

**Figure 10:**
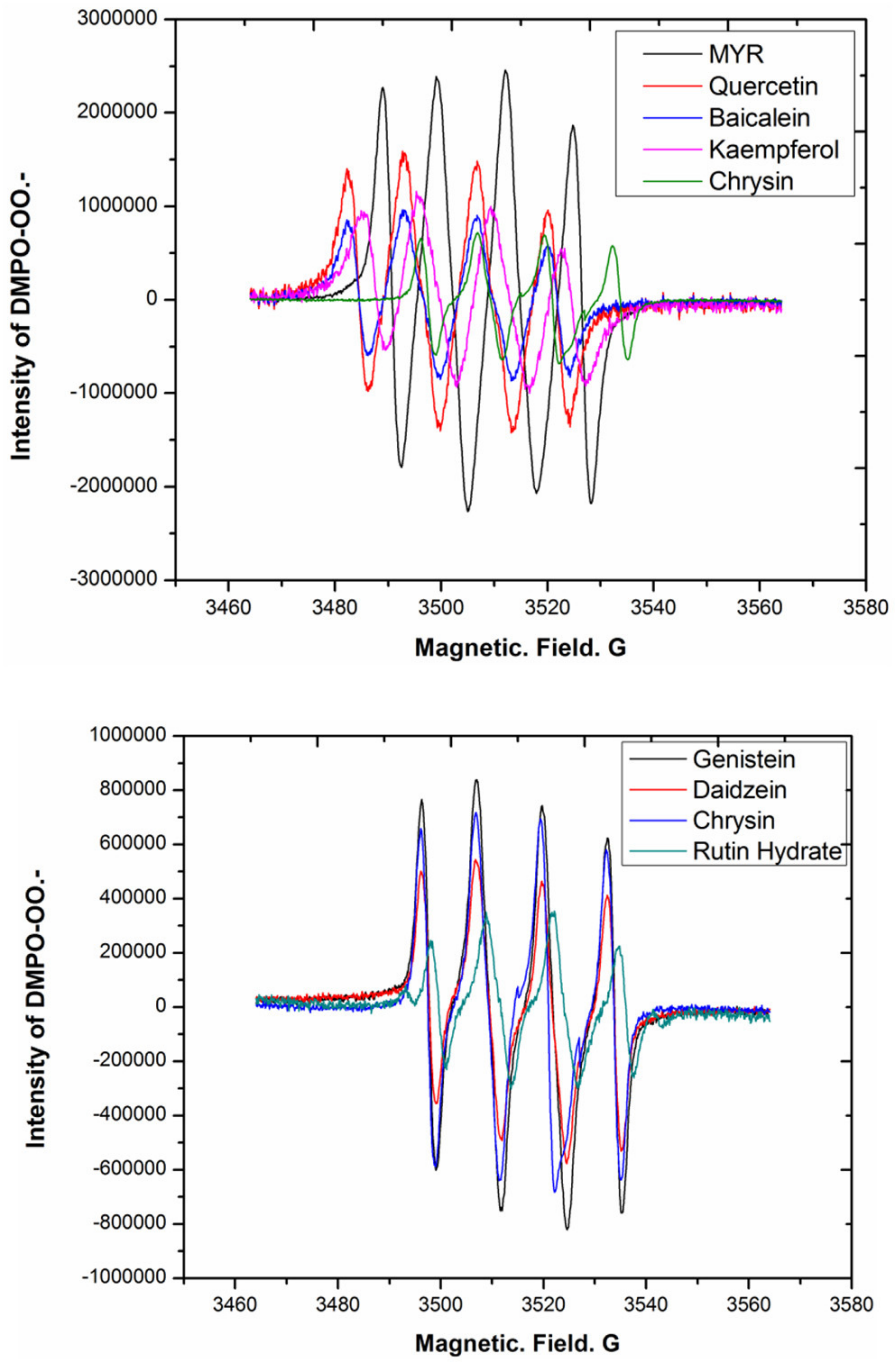
EPR spectra of DHM analogs autoxidized in an air-saturated alkaline solution.

Since DHM generates O_2_^−^ in an oxygenated alkaline solution, its analogs MYR, quercetin, daidzein, genistein, chrysin, baicalein, rutin hydrate, and kaempferol were also considered as potential ligands for metal complexes based on the multi-hydroxyl moieties attached to their molecular backbones (Figure 9). Thus, their behaviors in an air-saturated alkaline solution were investigated by EPR. All of these bioflavonoids originate from natural products and were tested at the same concentration in an air-saturated alkaline solution, then observed by EPR with DMPO (Figure 10). We found that the intensities of the DMPO-OO–adduct signal could be correlated to the location and/or number of phenolic hydroxyl groups or their configurations.

As shown in Table 1, the more phenolic hydroxyl groups are attached to the molecular skeleton, the higher the signal strength of the DMPO-OO–adduct in general. Comparing DHM with MYR, which have the same number of hydroxyl groups, the adduct signal was stronger for MYR under the same conditions. This indicated that molecular configuration contributes to the capacity for generating superoxide anions, potentially because of the transformation of DHM into MYR in the presence of O_2_^−^. For the flavonoids with three hydroxyl moieties, baicalein showed a higher adduct signal than genistein. At the same time, chrysin with two hydroxyl groups at the 5,7-positions of the molecular skeleton exhibited a higher DMPO-OO–adduct signal than daidzein. This indicated that molecular skeletons rich with electrons can promote the capacity for superoxide anion generation because of the +C effect from the hydroxyl functional groups. Rutin hydrate, composed of quercetin with rutinoside, was a particular example, as the adduct signal was markedly weaker than that of quercetin. These experimental results confirmed that the synthesis of bioflavonoid–metal complexes should exclude molecular oxygen from the reaction solution and employ fewer electronegative metal ions. Further investigations on work related to this issue is ongoing in our laboratory.

**Table 1:** Intensity of DMPO-OO–adduct EPR signal related to the characterization of bioflavonoids.

**Entry**	**Bioflavonoids**	**Characterization**	**Intensity of**
			**DMPO-OO–adduct^**a**^**
1	Dihydromyricetin	3, 5, 7, 3′, 4′, 5′ -OH	1827117.71
2	Myricetin	3, 5, 7, 3′, 4′, 5′ -OH	2383714.06
3	Quercetin	3, 5, 7, 3′, 4′ -OH	1576987.37
4	Kaempferol	3, 5, 7, 4′ -OH	1148836.33
5	Chrysin	5, 7 -OH	717476.37
6	Baicalein	5, 6, 7 -OH	952788.229
7	Daidzein	7, 4′ -OH	544170.9081
8	Genistein	5, 7, 4′ -OH	838478.55
9	Rutin hydrate	5, 7, 3′, 4′ -OH	349361.2188

a *Typical signals of DMPO-OO^−^ adduct performed to be maximum in value*.

## Conclusion

The general synthesis of bioflavonoid–metal complexes involves dissolving bioflavonoids in an air-saturated alkaline solution to undergo the deprotonation of phenolic hydroxyl groups and chelation with metals. However, because most bioflavonoids are unstable in air-saturated alkaline solutions, the general synthetic procedure may be inadequate. In this study, DHM as a representative bioflavonoid was selected as a potent ligand to synthesize DHM–M(II) complexes following the general procedure. The metal complexes were shown to be consistent with previous examples through spectroscopic analyses and evaluation of antioxidant capacity. Furthermore, the complexes were hydrolyzed to observe the stability of DHM via HPLC under acidic conditions. Surprisingly, many unknown absorption peaks with different intensities were observed at 254 nm in the LC spectra, indicating that the molecular structure of DHM was damaged during the synthesis of the DHM–M(II) complexes. This caused us to question the employed general synthetic procedure. To address this, the synthetic conditions and their effects on the DHM stability were investigated by UV-vis spectroscopy and HPLC. More electronegative metal ions and the solution alkalinity were shown to affect the DHM stability in an air-saturated solution and were thus regarded as probable causes of the observed damage to DHM using the general procedure. Furthermore, considering the content of MYR in the air-saturated alkaline solution of DHM, a pathway for the transformation of DHM into MYR via interaction with O_2_^−^ was proposed, involving a C3–H atom of DHM abstracted by O_2_^−^. The DHM analogs MYR, quercetin, daidzein, genistein, chrysin, baicalein, rutin hydrate, and kaempferol as potential bioflavonoid–metal complex ligands were also dissolved in air-saturated alkaline solutions and observed by EPR to generate O_2_^−^ at different capacities. These differences are possibly associated with the number and location of the multi-hydroxyl moieties attached to their molecular skeleton or their configurations. Therefore, the general synthetic procedure for bioflavonoid–metal complexes using a transition metal ion and air-saturated alkaline solution was shown to require improvement.

## Data Availability Statement

The raw data supporting the conclusions of this article will be made available by the authors, without undue reservation.

## Author Contributions

YY and SC were in charge of designing the experiments and writing the manuscript. YY, LH, YW, and MZ performed experiments. All authors contributed to the article and approved the submitted version.

## Conflict of Interest

The authors declare that the research was conducted in the absence of any commercial or financial relationships that could be construed as a potential conflict of interest.

## References

[B1] Amirhossein A. Shadboorestan A. (2016). Oxidative stress and cancer; the role of hesperidin, a citrus natural bioflavonoid, as a cancer chemoprotective agent. Nutr. Cancer 68, 29–39. 10.1080/01635581.2015.1078822 26381129

[B2] Branchini B. R. Behney C. E. Southworth T. L. Fontaine D. M. Gulick A. M. Vinyard D. J.. (2015). Experimental support for a single electron-transfer oxidation mechanism in firefly bioluminescence. J. Am. Chem. Soc. 137, 7592–7595. 10.1021/jacs.5b03820 26057379

[B3] Chen X. M. Tait A. R. Kitts D. D. (2017). Flavonoid composition of orange peel and its association with antioxidant and anti-inflammatory activities. Food Chem. 218, 15–21. 10.1016/j.foodchem.2016.09.016 27719891

[B4] Dai W. Bi J. Li F. Wang S. Huang X. Meng X.. (2019). Antiviral efficacy of flavonoids against enterovirus 71 infection *in vitro* and in newborn mice. Viruses 11:625. 10.3390/v11070625 31284698 PMC6669683

[B5] Gençkal H. M. Erkisa M. Alper P. Sahin S. Ulukaya E. Ari F. (2020). Mixed ligand complexes of Co (II), Ni (II) and Cu (II) with quercetin and diimine ligands: synthesis, characterization, anti-cancer and anti-oxidant activity. *J. Biol. Inorg*. Chem. 25, 161–177. 10.1007/s00775-019-01749-z 31832781

[B6] Imran M. Rauf A. Abu-Izneid T. Nadeem M. Shariati M. A. Khan I. A.. (2019). Luteolin, a flavonoid, as an anticancer agent: a review. *Biomed*. Pharmacother. 112:108612. 10.1016/j.biopha.2019.108612 30798142

[B7] Jabeen E. Janjua N. K. Ahmed S. Murtaza I. Ali T. Hameed S. (2017). Radical scavenging propensity of Cu^2+^, Fe^3+^ complexes of flavonoids and in-vivo radical scavenging by Fe^3+^-primuletin. Spectrochim. Acta Part A Mol. Biomol. Spectrosc. 171, 432–438. 10.1016/j.saa.2016.08.035 27572737

[B8] Khater M. Ravishankar D. Greco F. Osborn H. M. (2019). Metal complexes of flavonoids: their synthesis, characterization and enhanced antioxidant and anticancer activities. *Future Med*. Chem. 11, 2845–2867. 10.4155/fmc-2019-0237 31722558

[B9] Lalani S. Poh C. L. (2020). Flavonoids as antiviral agents for Enterovirus A71 (EV-A71). Viruses 12:184 10.3390/v12020184 PMC7077323 32041232

[B10] Lani R. Hassandarvish P. Shu M. H. Phoon W. H. Chu J. J. H. Higgs S.. (2016). Antiviral activity of selected flavonoids against Chikungunya virus. Antiviral. Res. 133, 50–61. 10.1016/j.antiviral.2016.07.009 27460167

[B11] Li X. Liu J. Lin J. Wang T. Huang J. Lin Y.. (2016). Protective effects of dihydromyricetin against ∙OH-induced mesenchymal stem cells damage and mechanistic chemistry. Molecules 21:604. 10.3390/molecules21050604 27171068 PMC6274564

[B12] Liang J. Wu J. Wang F. Zhang P. F. Zhang X. M. (2019). Semaphoring 4D is required for the induction of antioxidant stress and anti-inflammatory effects of dihydromyricetin in colon cancer. *Int*. Immunopharmac. 67, 220–230. 10.1016/j.intimp.2018.12.025 30562683

[B13] Liu K. J. Jiang J. J. Ji L. L. Shi X. Swartz H. M. (1996). An HPLC and EPR investigation on the stability of DMPO and DMPO spin adducts *in vivo*. Res. Chem. Intermediat. 22, 499–509. 10.1163/156856796X00700

[B14] Madunić J. Madunić I. V. Gajski G. Popić J. Garaj-Vrhovac V. (2018). Apigenin: a dietary flavonoid with diverse anticancer properties. Cancer Lett. 413, 11–22. 10.1016/j.canlet.2017.10.041 29097249

[B15] Maitera O. N. Louis H. Barminas J. T. Akakuru O. U. Boro G. (2018). Synthesis and characterization of some metal complexes using herbal flavonoids. Nat. Prod. Chem. Res. 6:314 10.4172/2329-6836.1000314

[B16] Qian J. Z. Wang B. C. Fan Y. Tan J. Yang X. (2015). QSAR study of flavonoid-metal complexes and their anticancer activities. J. Struct. Chem. 56, 338–345. 10.1134/S0022476615020195

[B17] Raffa D. Maggio B. Raimondi M. V. Plescia F. Daidone G. (2017). Recent discoveries of anticancer flavonoids. Eur. J. Med. Chem. 142, 213–228. 10.1016/j.ejmech.2017.07.034 28793973

[B18] Samsonowicz M. Regulska E. Kalinowska M. (2017). Hydroxyflavone metal complexes-molecular structure, antioxidant activity and biological effects. Chem. Biol. Interact. 273, 245–256. 10.1016/j.cbi.2017.06.016 28625490

[B19] Silva W. M. B. Oliveira Pinheiro S. Alves D. R. Menezes J. E. S. A. Magalhães F. E. A. Silva F. C. O.. (2020). Synthesis of Quercetin-metal complexes, *in vitro* and *in silico* anticholinesterase and antioxidant evaluation, and *in vivo* toxicological and anxiolitic activities. Neurotox. Res. 37, 893–903. 10.1007/s12640-019-00142-7 31853730

[B20] Spagnuolo C. Moccia S. G. Russo L. (2018). Anti-inflammatory effects of flavonoids in neurodegenerative disorders. Eur. J. Med. Chem. 153, 105–115. 10.1016/j.ejmech.2017.09.001 28923363

[B21] Tamayo L. V. Gouvea L. R. Sousa A. C. Albuquerque R. M. Teixeira S. F. de Azevedo R. A. (2016). Copper (II) complexes with naringenin and hesperetin: cytotoxic activity against A 549 human lung adenocarcinoma cells and investigation on the mode of action. Bio. Metals 29, 39–52. 10.1007/s10534-015-9894-0 26582127

[B22] Thangavel P. Viswanath B. Kim S. (2018). Synthesis and characterization of kaempferol-based ruthenium (II) complex: a facile approach for superior anticancer application. Mater. Sci. Eng. 89, 87–94. 10.1016/j.msec.2018.03.020 29752123

[B23] Tu L. Y. Pi J. Jin H. Cai J. Y. Deng S. P. (2016). Synthesis, characterization and anticancer activity of kaempferol-zinc(II) complex. Bioorg. Med. Chem. Lett. 26, 2730–2734. 10.1016/j.bmcl.2016.03.091 27080177

[B24] Uivarosi V. Munteanu A. C. Sharma A. Singh Tuli H. (2019). “Metal complexation and patent studies of flavonoid,” in Current Aspects of Flavonoids: Their Role in Cancer Treatment, H. Singh Tuli (Singapore: Springer), 39–77.

[B25] Wu Y. Xiao Y. Yue Y. Zhong K. Zhao Y. Gao H. (2020). A deep insight into mechanism for inclusion of 2R, 3R-dihydromyricetin with cyclodextrins and the effect of complexation on antioxidant and lipid-lowering activities. Food Hydrocolloid 103:105718 10.1016/j.foodhyd.2020.105718

[B26] Zhang H. Tsao R. (2016). Dietary polyphenols, oxidative stress and antioxidant and anti-inflammatory effects. Curr. Opin. Food Sci. 8, 33–42. 10.1016/j.cofs.2016.02.00232607320

