# Corrigendum: Evaluation of General Synthesis Procedures for Bioflavonoid–Metal Complexes in Air-Saturated Alkaline Solutions

**DOI:** 10.3389/fchem.2020.594058

**Published:** 2020-11-11

**Authors:** Yuanyong Yao, Meng Zhang, Laibing He, Yunyang Wang, Shixue Chen

**Affiliations:** Tongren Key Laboratory for Modernization Research, Development and Utilization of Traditional Chinese Medicine and National Medicine, School of Material and Chemical Engineering, Tongren University, Tongren, China

**Keywords:** dihydromyricetin, flavonoids, bioflavonoid–metal complexes, alkaline conditions, superoxide anion, HPLC, UV-visible spectrophotometer, transition metal ions

In the original article, there was a mistake in the legends for **Figures 3**, **4**, **7**, and **8** as published. In these Figures, marks such as a, b, c, d, e, f, and g have not been explained. The updated legends appear below.

**Figure 3. (A)** UV-vis spectra and **(B)** main peak positions of DHM refluxed for different times. (a) DHM; (b–g) DHM refluxing for 30, 60, 90, 120, 150, and 180 min.

**Figure 4. (A)** UV-vis spectra and **(B)** main peak positions of DHM stirred for different times at pH = 8.2. (a) DHM; (b–g) DHM stirring for 1, 3, 5, 10, 20, and 30 min in pH 8.2.

**Figure 7**. EPR spectra of DHM in air-, nitrogen-, and oxygen-saturated alkaline solutions. (a) DHM only; (b) DHM + DMPO; (c–e) DHM + DMPO, in nitrogen-, air-, oxygen-saturated alkaline solution.

**Figure 8**. EPR spectra of superoxide-anion radical generation from DMSO reacted with a base in the presence of oxygen. (a) DMSO + DMPO + O_2_; (b) Na^+^PhO^−^ + DMPO + O_2_; (c) Na^+^PhO^−^+O_2_; (d) DMSO + Na^+^PhO^−^ + O_2_; (e) DMSO + Na^+^PhO^−^+ DMPO + O_2._

The authors apologize for this error and state that this does not change the scientific conclusions of the article in any way. The original article has been updated.

