# Supplementary material for: Evaluation of General Synthesis Procedures for Bioflavonoid–Metal Complexes in Air-Saturated Alkaline Solutions
Source: Front Chem. 2020 Aug 5;8:589. doi: 10.3389/fchem.2020.00589 (PMC7419984; doi:10.3389/fchem.2020.00589)
Supplement: Supplementary file 1 [file Data_Sheet_1.docx]

Supplementary Material

Disclosure of general procedures failed for synthesis of bioflavonoids metal complexes

Yuanyong Yao^1*^, Meng Zhang^1^, Laibing He^1^, Yunyang Wang^1^, Shixue Chen^1*^

^1^Institute of applied chemistry; School of material and chemical engineering, Tongren university, Tongren, (P.R) China.

*** Correspondence:**

Yuanyong Yao
E-mail: chyyyy@gztrc.edu.cn (Y. Y.);

Shixue Chen

E-mail: [Tongrencsx01@126.com](mailto:Tongrencsx01@126.com) (C. S).

Keywords: dihydromyricetin, bioflavonoids, metal complexes, alkaline solution, synthesis.







**Supplementary Figure 1.** DHM metal complexes such as DHM-Co(II), DHM-Cu(II), DHM-Fe(II), DHM-Zn(II), DHM-Ni(II) complexes identified by FT-IR technique.

**Supplementary Table 1**. The main infrared absorption frequency of DHM-M(II) complexes.

| Compounds | Compounds IR, v(cm^-1^) | | | |
| --- | --- | --- | --- | --- |
|  | v（O-H） | v(C=O) | v（C-O-C） | v(M-O) |
| DHM | 3591 | 1654 | 1274、1139、1081、1029 | — |
| DHM-Co(II) | 3514 | 1604 | 1262、1174、1090 | 659 |
| DHM-Ni(II) | 3600 | 1636 | 1262、1168、1092 | 657 |
| DHM-Fe(II) | 3577 | 1624 | 1279、1104 | 626 |
| DHM-Zn(II ) | 3605 | 1659 | 1279、1174、1046 | 651 |
| DHM-Cu(II) | 3562 | 1620 | 1274、1176、1089、1029 | 648 |

**Supplementary Table 2**. Element analyses of DHM-Co(II), DHM-Cu(II), DHM-Fe(II), DHM-Zn(II), DHM-Ni(II) complexes

| Sample | Chemical Formula | Elem.Anal | Found | Structure |
| --- | --- | --- | --- | --- |
| DHM-Cu(II)^a^ | C_19_H_17_CuO_12_ | C, 45.56;  H, 3.42;  Cu, 12.69;  O, 38.33 | C, 45.59;  H, 3.39; |  |
| DHM-Cu(II)^b^ | C_19_H_16_CuO_12_ | C, 45.65;  H, 3.23;  Cu, 12.71;  O, 38.41 |  |  |
| DHM-Co(II)^a^ | C_19_H_17_CoO_12_ | C, 45.98;  H, 3.45; Co,11.88;  O, 38.69 | C, 45.51;  H, 3.40; |  |
| DHM-Co(II)^b^ | C_19_H_16_CoO_12_ | C, 46.08;  H, 3.26;  Co, 11.90;  O, 38.77 |  |  |
| DHM-Fe(II)^a^ | C_19_H_17_FeO_12_ | C, 46.27;  H, 3.47;  Fe, 11.32;  O, 38.93 | C, 46.31;  H, 3.53 |  |
| DHM-Fe(II)^b^ | C_19_H_16_FeO_12_ | C, 46.37;  H, 3.28;  Fe, 11.35;  O, 39.01 |  |  |
| DHM-Zn(II)^a^ | C_19_H_17_ZnO_12_ | C, 45.39;  H, 3.41;  Zn, 13.01;  O, 38.19 | C, 45.33;  H, 3.47 |  |
| DHM-Zn(II)^b^ | C_19_H_16_ZnO_12_ | C, 45.49;  H, 3.21;  Zn, 13.03;  O, 38.27 |  |  |
| DHM-Ni(II)^a^ | C_19_H_17_NiO_12_ | C, 46.01;  H, 3.45;  Ni, 11.83;  O, 38.71 | C, 46.11;  H, 3.48 |  |
| DHM-Ni(II)^b^ | C_19_H_16_NiO_12_ | C, 46.10;  H, 3.26;  Ni, 11.86;  O, 38.79 |  |  |

Note:^a^ the structure involving in formation of six-members ring; ^b^ the structure involving in formation of five-members ring.


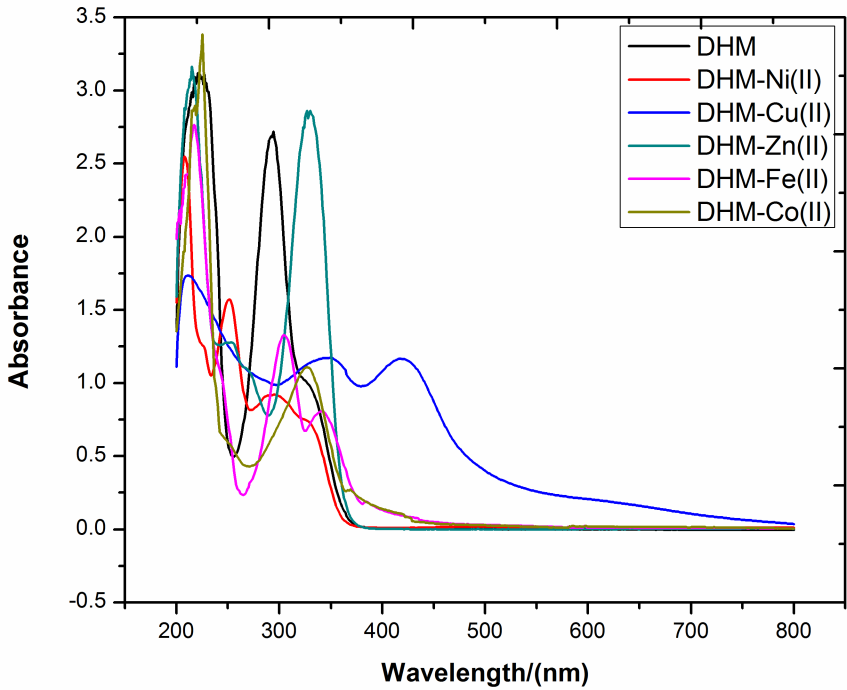


**Supplementary Figure 2.** UV-visible spectra of DHM-Co(II), DHM-Cu(II), DHM-Fe(II), DHM-Zn(II), DHM-Ni(II) complexes


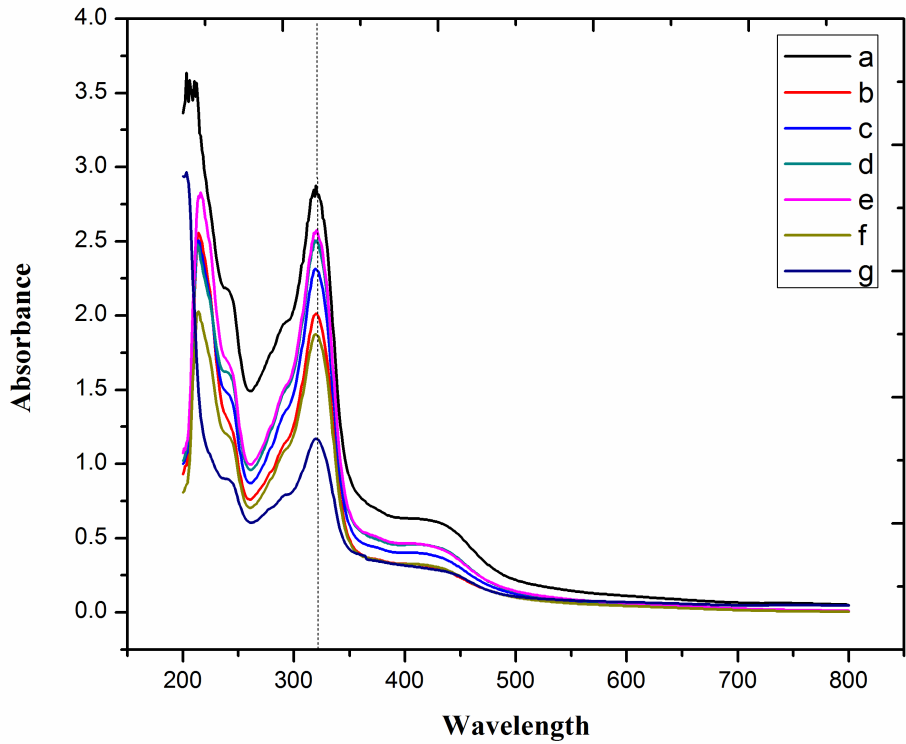


# **Supplementary Figure 3.** Evaluation of DHM-Co(II), DHM-Cu(II), DHM-Fe(II), DHM-Zn(II), DHM-Ni(II) complexes in antioxidant capacity.

Note: (a) control group: autoxidation of pyrogallol in air-saturated alkaline solution. (b) autoxidation of pyrogallol + DHM(0.01mg/mL). (c) autoxidation of pyrogallol + DHM-Cu(II)(0.01mg/mL). (d) autoxidation of pyrogallol + DHM-Co(II)(0.01mg/mL), (e) autoxidation of pyrogallol + DHM-Ni(II)(0.01mg/mL), (f) autoxidation of pyrogallol + DHM-Fe(II)(0.01mg/mL), and (g) autoxidation of pyrogallol + DMY-Zn(II) (0.01mg/mL)

**Supplementary Table 3.** The inhibition of DHM-M(II) complexes observed by UV-visible spectrometer in antioxidant capacity.

| Name | Concentration(mg/mL) | Absorbance | Inhibition(%) | Monitoring wavelength(nm) |
| --- | --- | --- | --- | --- |
| Pyrogallol autoxidation method | 2.00 | 2.848022110 | ----- | 325 |
| DHM | 0.01 | 2.016060810 | 29.21 | 325 |
| DHM-Cu(II) | 0.01 | 2.315064917 | 18.71 | 325 |
| DHM-Co(II) | 0.01 | 2.505149978 | 12.04 | 325 |
| DHM-Ni(II) | 0.01 | 2.574139855 | 9.62 | 325 |
| DHM-Fe(II) | 0.01 | 1.873346552 | 34.22 | 325 |
| DHM-Zn(II) | 0.01 | 1.170381076 | 58.91 | 325 |


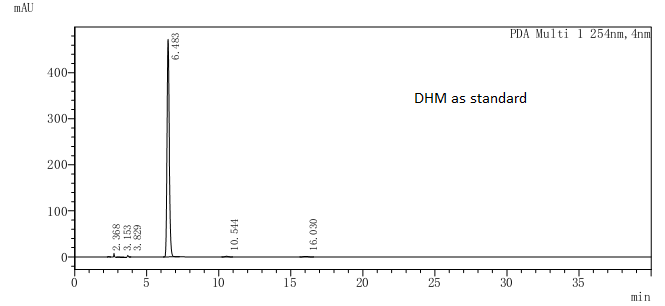


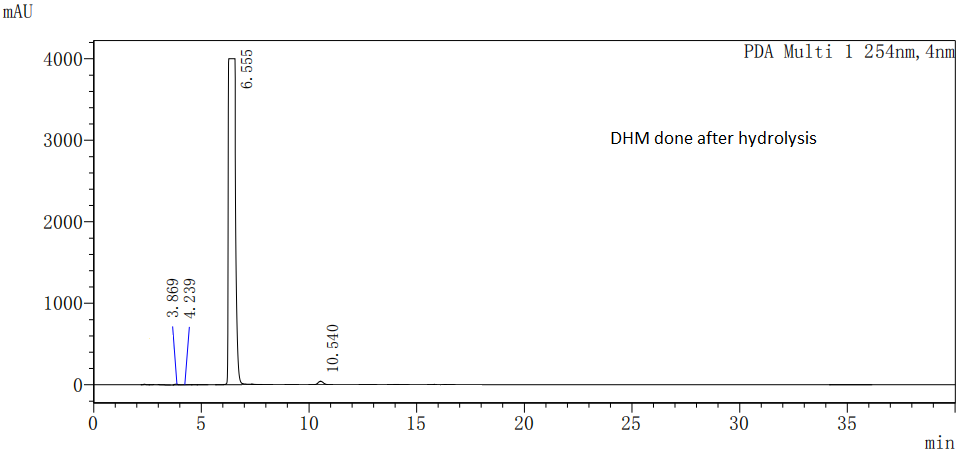


**
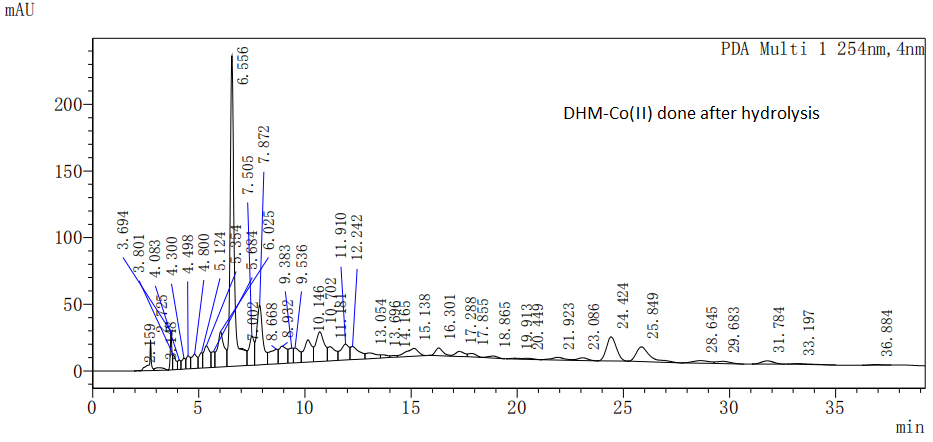
**

**
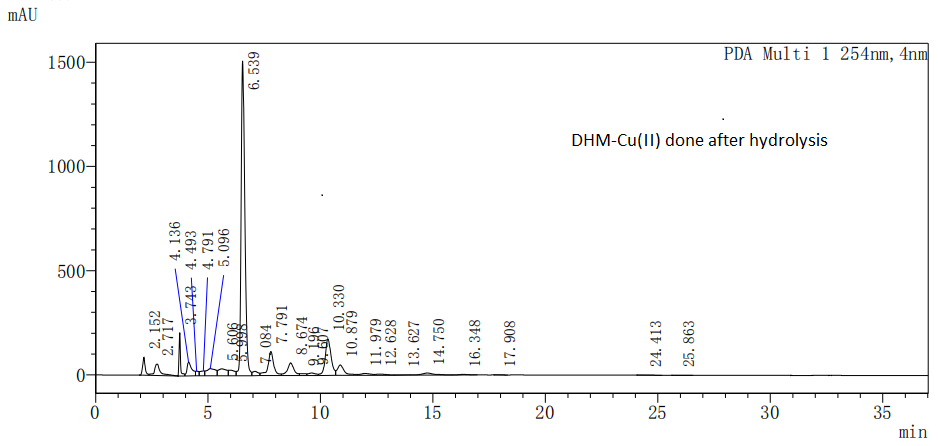
**

**
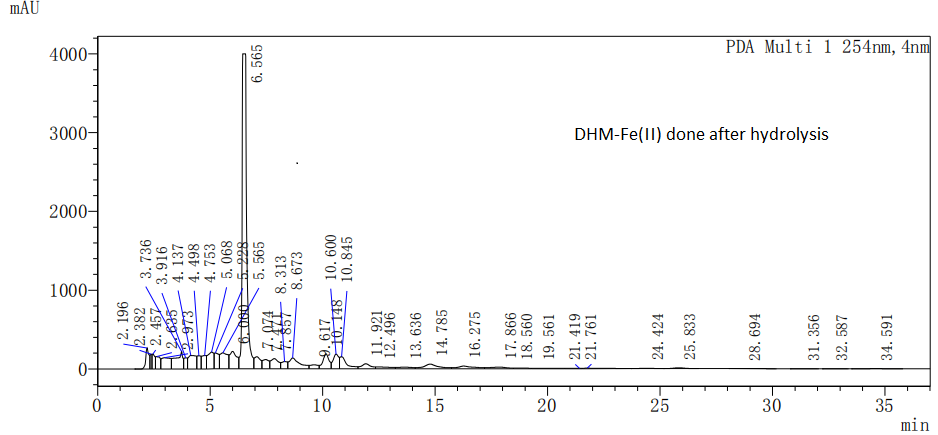
**

**
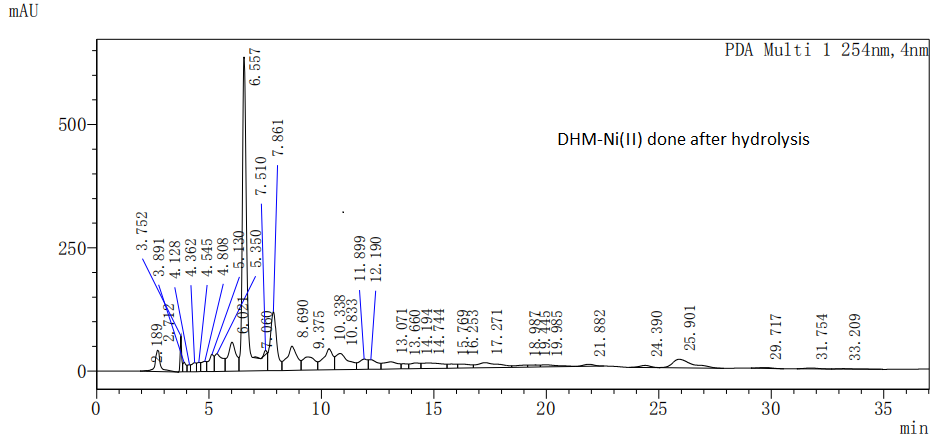
**

**
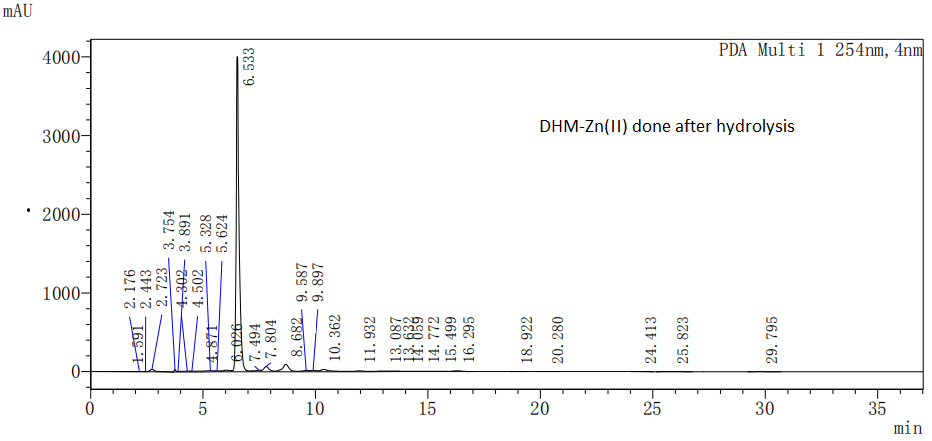
**

**Supplementary Figure 4.** DHM-M(II) after hydrolysis made under acidic conditions observed by HPLC, as comparing to free DHM.

**Diagnosis by HPLC and chromatographic conditions**

**Chromatographic conditions:** the chromatography was performed on a Hypersil BDS C18 column (4.6mm*200mm, 5um). The mobile phase consisted of acetonitrile (MeCN, A) and water containing 0.1% (v/v) phosphoric acid (B). The elution condition was A-B (24:76, v/v), The mobile phase was filtered through a 0.45um Millipore filter and degassed before use. The flow rate was 1.0 mL/min. The detector wavelengths were set at 254 nm. The injection volume was 5ul and the column temperature was set at 25℃.

The calibration standard solution of DHM was prepared at a concentration of 10mg/mLin methanol. By taking 20uL, 40uL, 60uL, 80uL, 100uL from standard solution(10mg/mL) into flask, respectively, the external standard solutions were diluted into final concentration of 0.2mg/mL, 0.4 mg/mL, 0.6 mg/mL, 0.8 mg/mL, 1.0 mg/mL in methanol. All solutions were protected from light and stored at 4℃.

**Working curve preparation**

The linearity was validated within the calibration range of 0.2–1.0mg/mL, the calibration curve were analyzed using linear regression equation, with 0.9998 in value of coefficient (R^2^). It is able to meet the acceptable criteria.


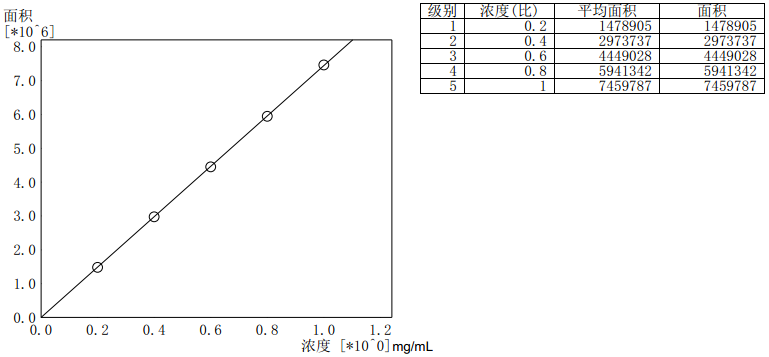


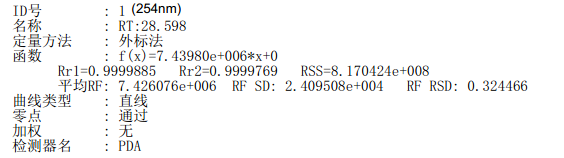


**Supplementary Figure 5.** Working curve of DHM

1. **In pH 7.4**


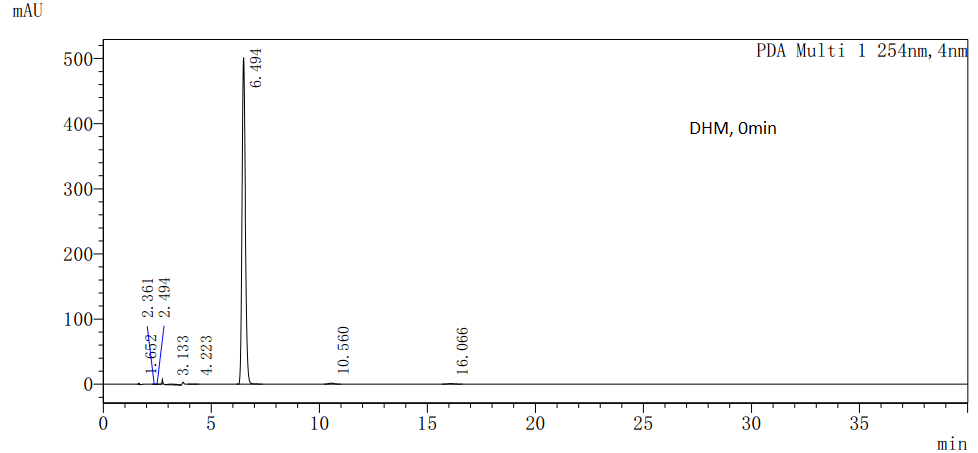

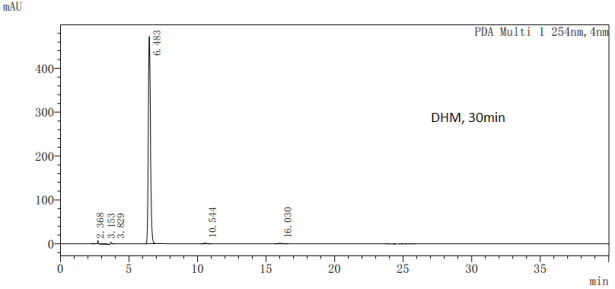


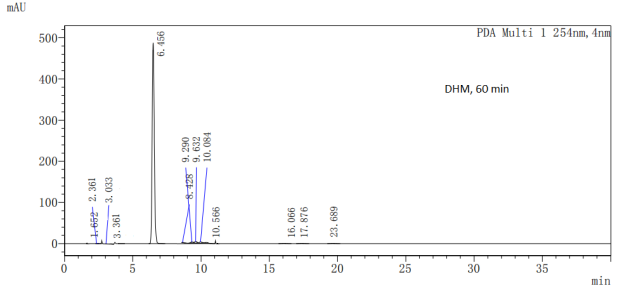

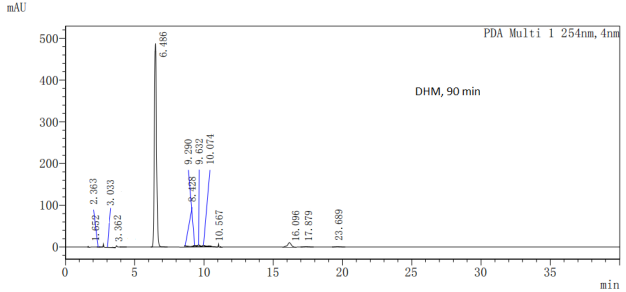

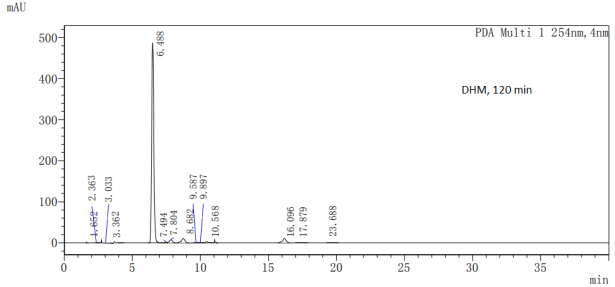


**Supplementary Figure 6.** HPLC monitoring alkalinity affected to DHM in quantitative analysis in pH 7.4

**Supplementary Table4.** The concentration of DHM detected by HPLC in pH 7.4

| Target | Reaction time (min) | Retention time (min) | Peak area | Peak height | C (mg/mL) |
| --- | --- | --- | --- | --- | --- |
| DHM | 0 | 6.494 | 5236310 | 501050 | 0.71 |
| DHM | 30 | 6.483 | 5191736 | 492517 | 0.70 |
| DHM | 60 | 6.456 | 5030139 | 488011 | 0.68 |
| DHM | 90 | 6.486 | 4887725 | 470939 | 0.66 |
| DHM | 120 | 6.488 | 4730139 | 451945 | 0.64 |

1. **In pH 8.2**


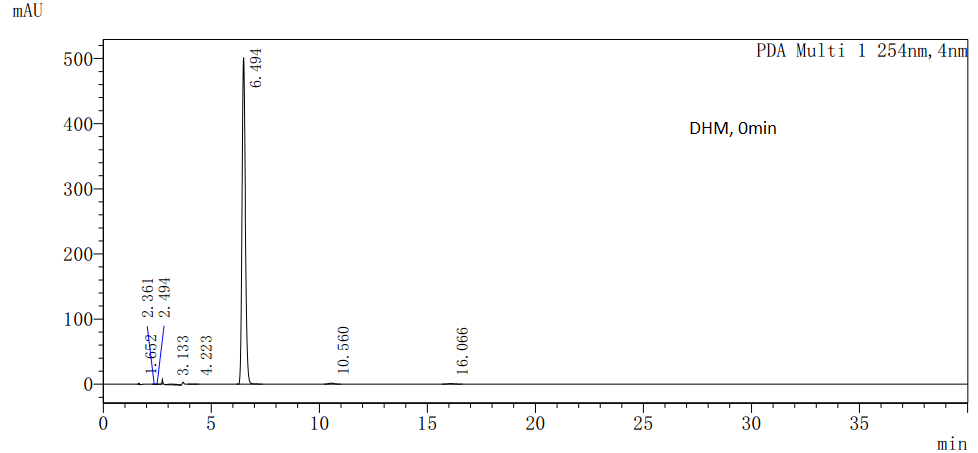
**
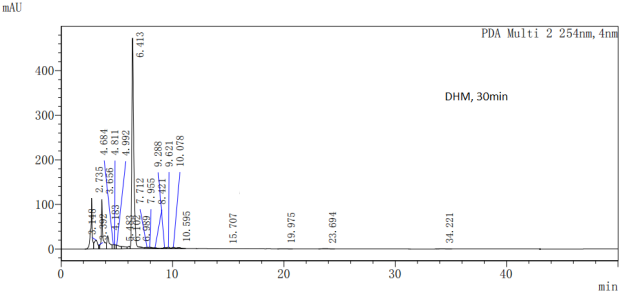
**

**
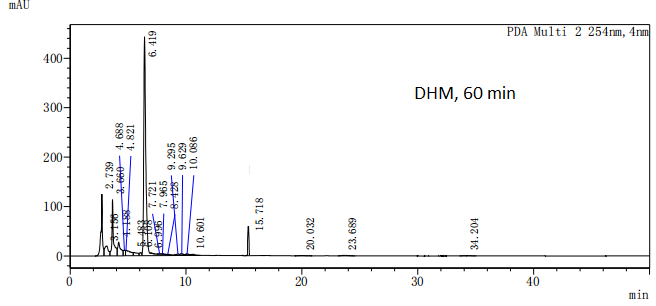

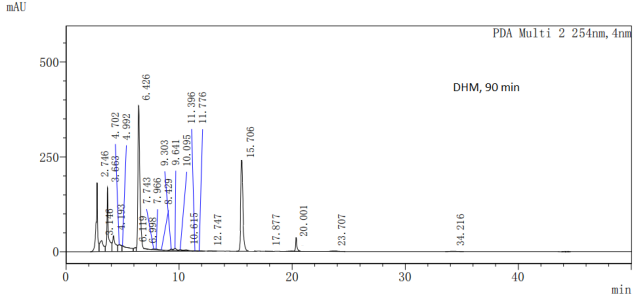
**

**
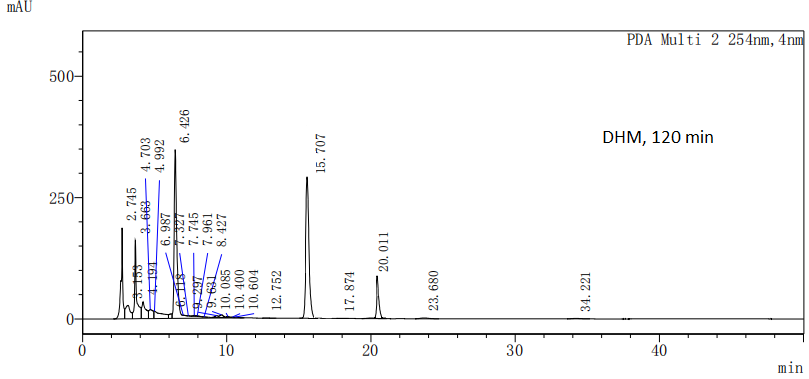
**

**Supplementary Figure 7.** HPLC monitoring alkalinity affected to DHM in quantitative analysis in pH 8.2

**Supplementary Table 5.** the concentration of DHM detected by HPLC in pH 8.2

| Target | Reaction time (min) | Retention time (min) | Peak area | Peak height | C (mg/mL) |
| --- | --- | --- | --- | --- | --- |
| DHM | 0 | 6.494 | 5236310 | 501050 | 0.71 |
| DHM | 30 | 6.413 | 4891939 | 478227 | 0.66 |
| DHM | 60 | 6.419 | 4774743 | 459418 | 0.64 |
| DHM | 90 | 6.426 | 4399844 | 390389 | 0.59 |
| DHM | 120 | 6.426 | 3998789 | 365887 | 0.54 |

1. **In pH 9.4**


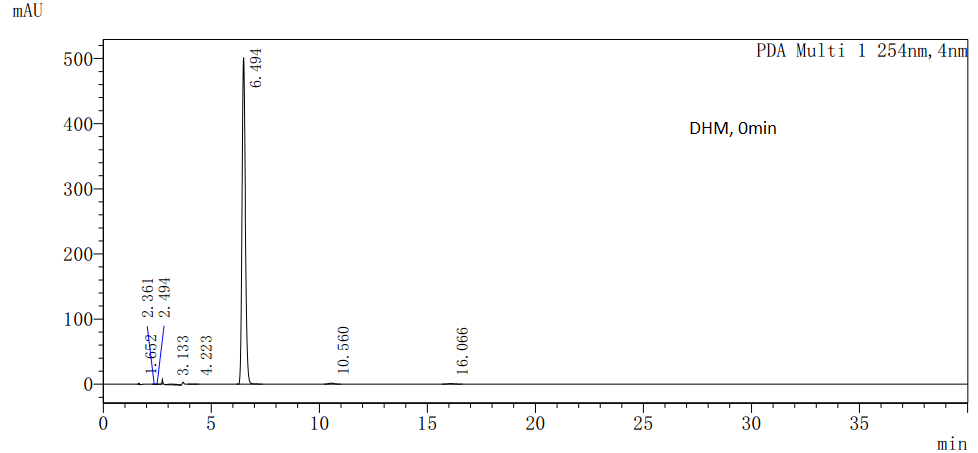
**
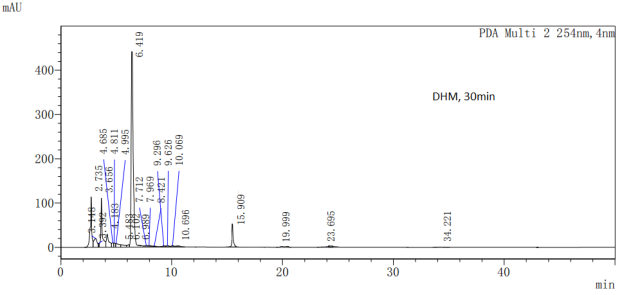
**

**
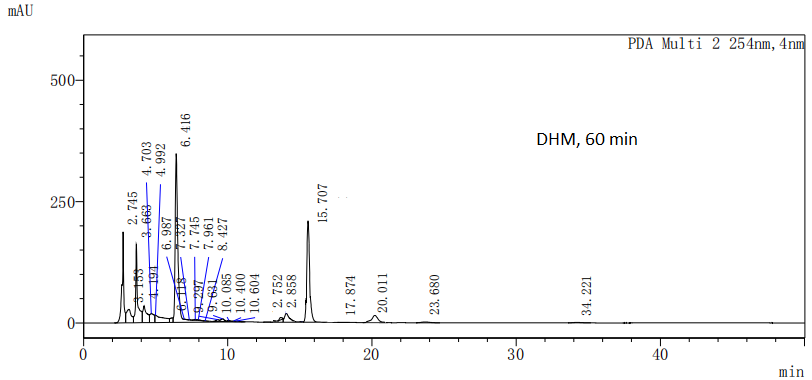

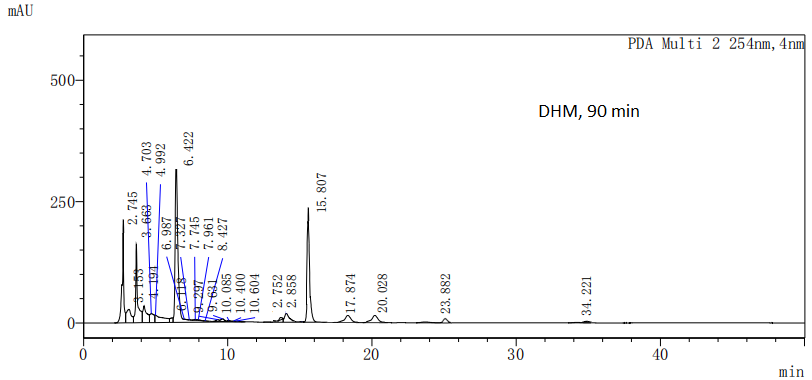
**

**
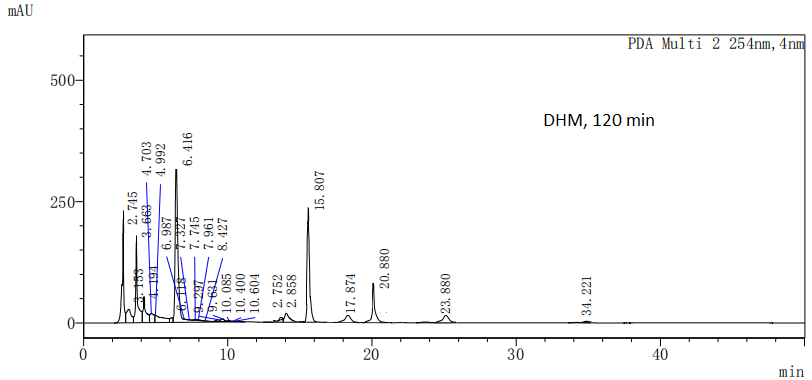
**

**Supplementary Figure 8.** HPLC monitoring alkalinity affected to DHM in quantitative analysis in pH 9.4

**Supplementary Table 6.** the concentration of DHM detected by HPLC in pH 9.4

| Target | Reaction time (min) | Retention time (min) | Peak area | Peak height | C (mg/mL) |
| --- | --- | --- | --- | --- | --- |
| DHM | 0 | 6.494 | 5236310 | 501050 | 0.71 |
| DHM | 30 | 6.419 | 4799736 | 458589 | 0.65 |
| DHM | 60 | 6.416 | 3938754 | 361081 | 0.53 |
| DHM | 90 | 6.422 | 3598706 | 331136 | 0.49 |
| DHM | 120 | 6.416 | 3099188 | 319055 | 0.42 |


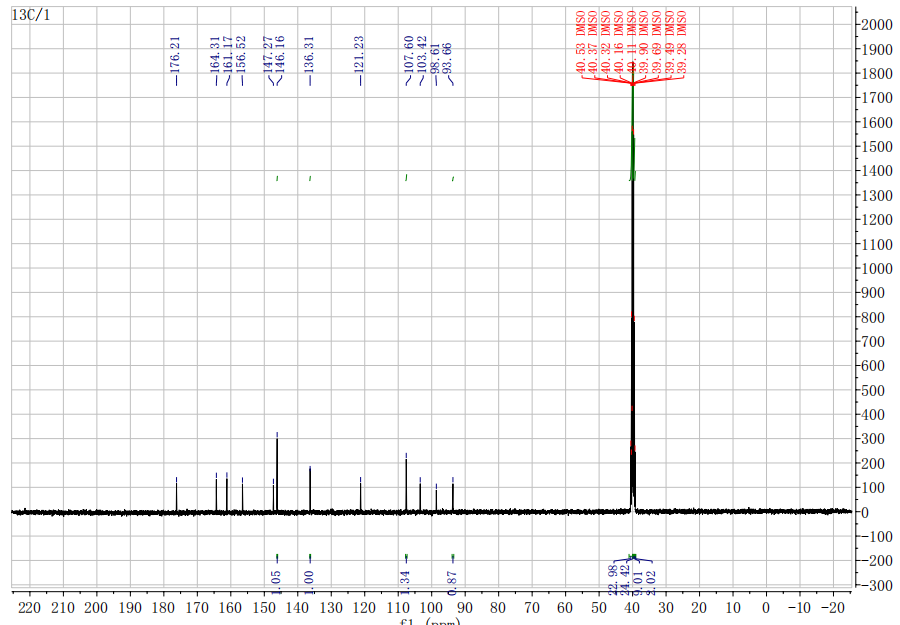


^13^C-NMR


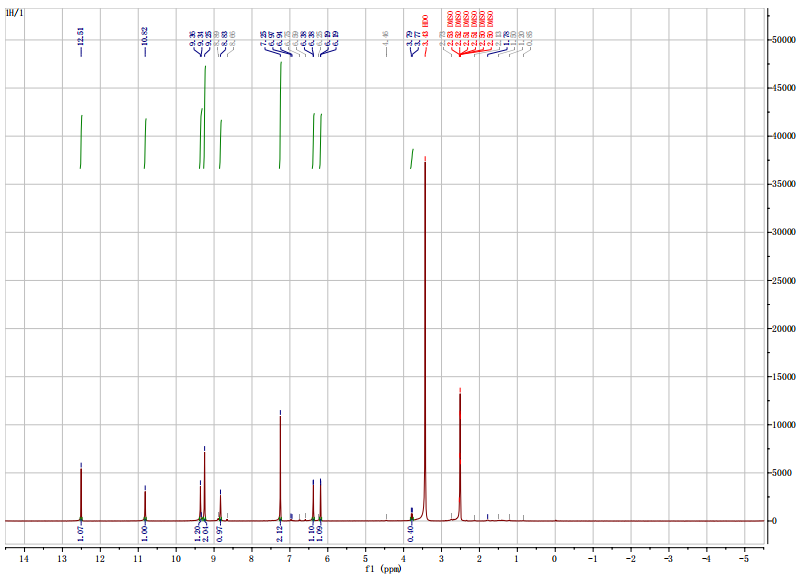


^1^H-NMR

ESI-Ms


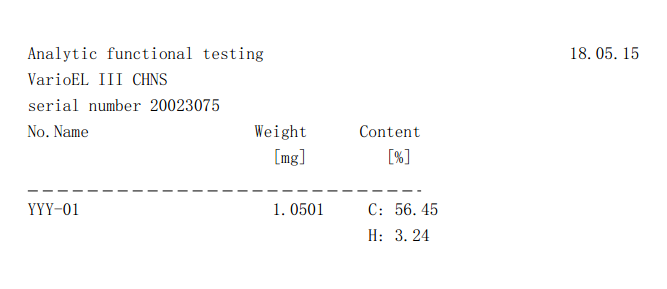


Element analyses

**Supplementary Figure 9.** The structure of myricetin identified by ^1^H- and ^13^C-NMR, ESI-MS, Element analyses


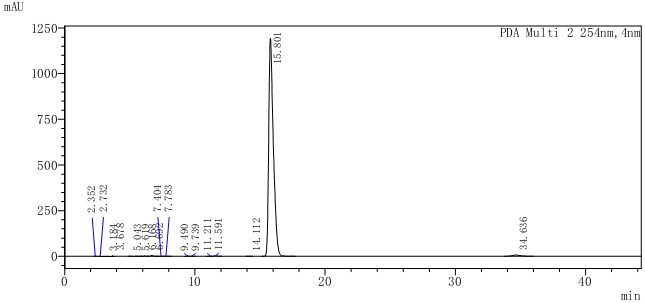


Note: chromatographic conditions : Hypersil BDS C_18_ (4.6*200mm, 5um), Elute: (Acetonitrile / Water (0.1% phosphoric acid) = 24 / 76), Flow rate = 1 ml/min, UV = 254nm.

**Supplementary Figure 10.** The component of myricetin separated by column chromatography and identified via HPLC


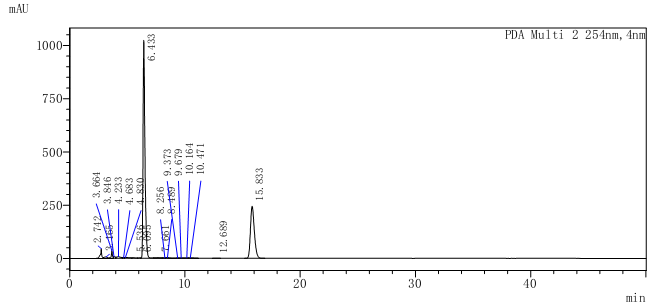


Note: chromatographic conditions : Hypersil BDS C_18_ (4.6*200mm, 5um), Elute: (Acetonitrile / Water (0.1% phosphoric acid) = 24 / 76), Flow rate = 1 ml/min, UV = 254nm.

**Supplementary Figure 11.** DHM transferred to the DMSO system observed by HPLC
